# Long-term Cardiovascular, Cerebrovascular, and Other Thrombotic Complications in COVID-19 Survivors: A Retrospective Cohort Study

**DOI:** 10.1093/cid/ciad469

**Published:** 2023-09-25

**Authors:** Jue Tao Lim, Wee Liang En, An Ting Tay, Deanette Pang, Calvin J Chiew, Benjamin Ong, David Chien Boon Lye, Kelvin Bryan Tan

**Affiliations:** Lee Kong Chian School of Medicine, Nanyang Technological University; National Centre for Infectious Diseases; National Centre for Infectious Diseases; Duke-NUS Graduate Medical School, National University of Singapore; Department of Infectious Diseases, Singapore General Hospital; Singapore Ministry of Health; Singapore Ministry of Health; National Centre for Infectious Diseases; Singapore Ministry of Health; Singapore Ministry of Health; Yong Loo Lin School of Medicine, National University of Singapore; Lee Kong Chian School of Medicine, Nanyang Technological University; National Centre for Infectious Diseases; Yong Loo Lin School of Medicine, National University of Singapore; Department of Infectious Diseases, Tan Tock Seng Hospital; Lee Kong Chian School of Medicine, Nanyang Technological University; Singapore Ministry of Health; Saw Swee Hock School of Public Health, National University of Singapore

**Keywords:** cardiovascular, cerebrovascular, thrombotic, complications, postacute sequelae of SARS-CoV-2 infection

## Abstract

**Background:**

Growing evidence suggests that some coronavirus disease 2019 (COVID-19) survivors experience a wide range of long-term postacute sequelae. We examined the postacute risk and burden of new-incident cardiovascular, cerebrovascular, and other thrombotic complications after severe acute respiratory syndrome coronavirus 2 (SARS-CoV-2) infection in a highly vaccinated multiethnic Southeast Asian population, during Delta predominance.

**Methods:**

This cohort study used national testing and healthcare claims databases in Singapore to build a cohort of individuals who had a positive SARS-CoV-2 test between 1 September and 30 November 2021 when Delta predominated community transmission. Concurrently, we constructed a test-negative control group by enrolling individuals between 13 April 2020 and 31 December 2022 with no evidence of SARS-CoV-2 infection. Participants in both groups were followed up for a median of 300 days. We estimated risks of new-incident cardiovascular, cerebrovascular, and other thrombotic complications using doubly robust competing-risks survival analysis. Risks were reported using 2 measures: hazard ratio (HR) and excess burden (EB) with 95% confidence intervals.

**Results:**

We included 106 012 infected cases and 1 684 085 test-negative controls. Compared with the control group, individuals with COVID-19 exhibited increased risk (HR, 1.157 [1.069–1.252]) and excess burden (EB, 0.70 [.53–.88]) of new-incident cardiovascular and cerebrovascular complications. Risks decreased in a graded fashion for fully vaccinated (HR, 1.11 [1.02–1.22]) and boosted (HR, 1.10 [.92–1.32]) individuals. Conversely, risks and burdens of subsequent cardiovascular/cerebrovascular complications increased for hospitalized and severe COVID-19 cases (compared to nonhospitalized cases).

**Conclusions:**

Increased risks and excess burdens of new-incident cardiovascular/cerebrovascular complications were reported among infected individuals; risks can be attenuated with vaccination and boosting.

## Teaser

In the 300 days following infection, SARS-CoV-2–infected individuals exhibited increased risk of new-incident cardiovascular and cerebrovascular complications compared with test-negative controls. Risks decreased with vaccination and boosting. Conversely, risks increased for hospitalized and severe COVID-19 cases, compared to nonhospitalized cases.

Severe acute respiratory syndrome coronavirus 2 (SARS-CoV-2) infection has been associated with not just pulmonary but also multisystemic involvement, including cardiovascular and cerebrovascular systems [1–3]. Direct viral invasion, inflammation, and immunological responses can potentially damage the myocardium, pericardium, and conduction system [1, 2]. Similar mechanisms have been posited for neurologic manifestations in patients after coronavirus disease 2019 (COVID-19), including postinfectious autoimmune responses, hypercoagulability, endotheliopathy, and direct neuroinvasion [3]. Persistence of such damage can result in long-term sequelae [2, 3].

Multiple large retrospective cohort studies have reported increased long-term incidence of cardiovascular, cerebrovascular, and other thrombotic complications in COVID-19 survivors up to 1 year postinfection [4–11]. The majority of these studies, however, were confined to earlier waves driven by ancestral SARS-CoV-2 strains [4–7, 9, 10]. Newer SARS-CoV-2 variants, such as Omicron, were associated with lower odds of prolonged symptoms at 3–6 months from infection compared with pre-Delta variants [12, 13]. Whether differences in symptomatology translate into reduced long-term sequelae is at present unclear. Furthermore, most studies either predated vaccination [7, 9, 10] or excluded vaccinated individuals [8]. Emerging evidence suggests that vaccination is associated with a reduced risk of major adverse cardiovascular/cerebrovascular events (MACE) after SARS-CoV-2 infection [14, 15] and reduced risk of persistent post-COVID-19 symptoms, known as long COVID [13]. A study conducted during emergence of the Delta variant, utilizing the US Veterans Affairs database, demonstrated some degree of protection with vaccination, though vaccinated individuals still had higher risk of postacute sequelae at 6 months, compared with contemporary uninfected controls [11]. Given the large and growing number of people with COVID-19, even a modestly increased risk postacute infection may translate into a substantial rise in overall disease burden, with significant consequences. An analysis early on in the pandemic estimated that long COVID might result in $2.6 trillion of cost, attributable to reductions in length as well as quality of life [16]; however, such assumptions may not hold in the current context of widely available vaccination and emergence of milder SARS-CoV-2 variants.

We retrospectively constructed a national cohort of individuals infected during a 3-month period when transmission of the Delta variant predominated in Singapore, a multiethnic Southeast Asian city-state. This cohort was compared against a test-negative control group comprising infection-naive individuals, and followed longitudinally to estimate the 300-day risk and excess burdens of new-incident prespecified cardiovascular, cerebrovascular, and other thrombotic complications.

## METHODS

### Study Setting and Databases

We used the national healthcare claims database to ascertain incidence of cardiovascular, cerebrovascular, and other thrombotic complications for infected cases and uninfected test-negative controls before and after SARS-CoV-2 infection, in a retrospective cohort study. Inpatient care is predominantly provided by public hospitals, which account for 77.8% of admissions; care is financed by reimbursement claims against a national medical savings scheme [17]. SARS-CoV-2 infection status (either positive polymerase chain reaction [PCR] or rapid antigen test [RAT]) was determined based on data collected from national databases maintained by the Ministry of Health (MOH), Singapore. Throughout the pandemic period, individuals with acute respiratory illness (ARI) were strongly encouraged via public health messaging to seek consultation for free SARS-CoV-2 testing, which was provided at all public primary care clinics (polyclinics) and Public Health Preparedness Clinics (PHPCs), a nationwide network of >1000 private general practitioner clinics [18]. Testing for SARS-CoV-2 (PCR/RAT) was compulsory for all individuals who presented with ARI symptoms to any healthcare provider, and positive cases were legally required to be notified to MOH [18]. Severity of initial infection was classified by care setting (mild disease: outpatient management only); hospitalized cases (intermediate severity); and severe disease, defined as hospitalized cases who additionally required oxygen supplementation or intensive care unit/high-dependency admission. All healthcare facilities notified MOH of hospitalized cases and severe infections [18].

### Vaccination Program and Waves of SARS-CoV-2 Transmission in Singapore

Two mRNA vaccines were approved under Singapore’s national vaccination program: BNT162b2 (Pfizer) and mRNA-1273 (Moderna) [19]. A complete vaccine regimen involved 2 doses 3–8 weeks apart. In September 2021, persons aged ≥60 years were recommended to receive a booster 6–9 months after the second dose. By the end of November 2021, 94% had received 2 doses and 24% had received a booster [20]. The Delta variant was first detected in April 2021, predominating community transmission (≥90% of sequenced cases on national genomic surveillance) by October 2021; Delta predominated up to January 2022 when Omicron BA.1/2 replaced Delta as the dominant strain [21].

### Cohort

**Figure 1.**
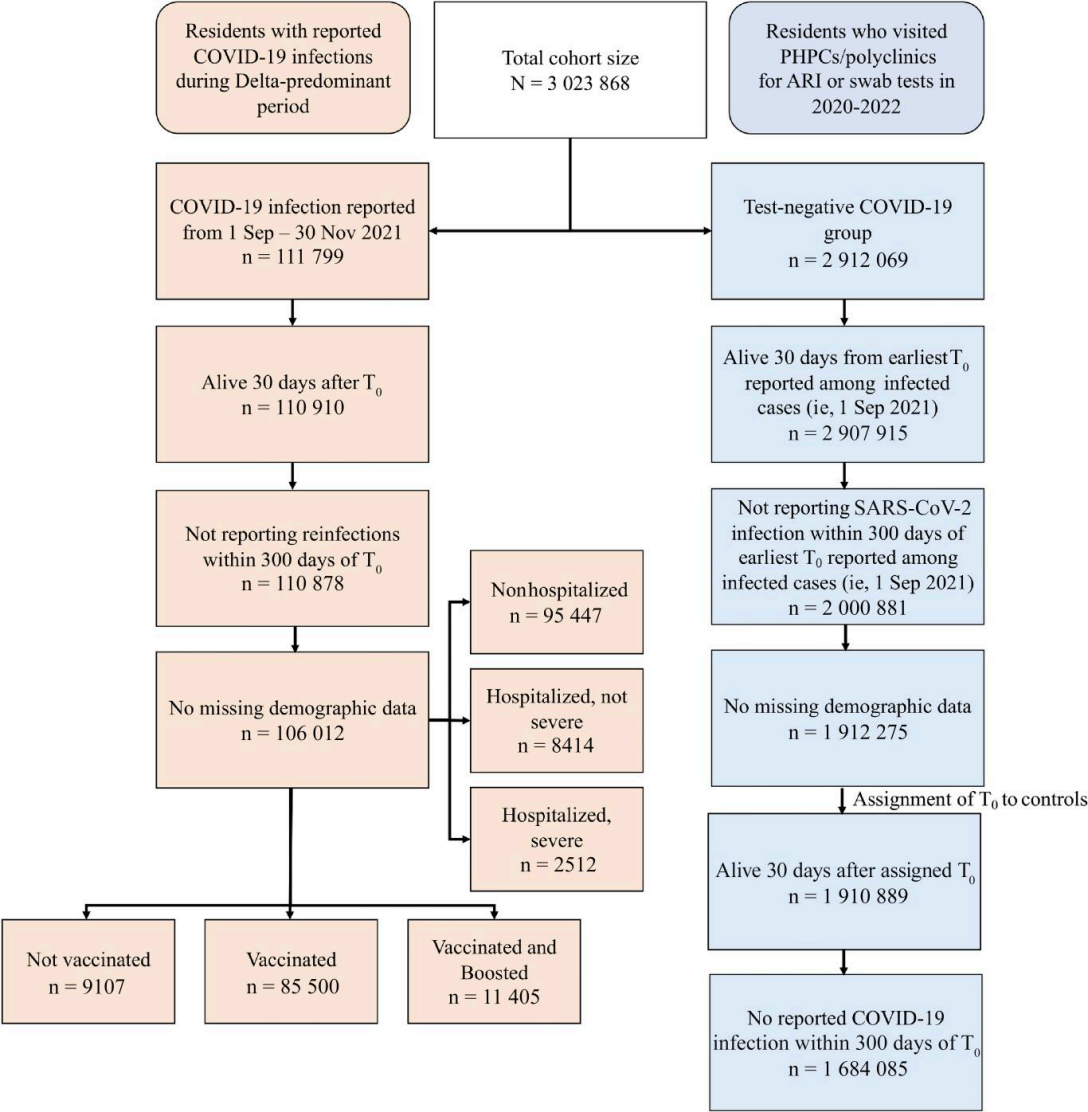
Flowchart of cohort construction. Abbreviations: ARI, acute respiratory illness; COVID-19, coronavirus disease 2019; PHPCs, Public Health Preparedness Clinics; SARS-CoV-2, severe acute respiratory syndrome coronavirus 2; T_0_, index date of infection.

A flowchart of cohort construction from participants enrolled between 1 September 2021 and 30 November 2022 is provided in Figure 1. We enrolled individuals who were older than 18 years and were Singapore citizens or permanent residents.

Cases were taken as those who were infected with SARS-CoV-2 for the first time during the period when transmission of the Delta variant predominated (1 September–30 November 2021), with the index date of infection (T_0_) taken as the date of first positive PCR/RAT. We excluded individuals who died within 30 days or had reinfections within 300 days of T_0_. Controls were taken as individuals who presented to primary care (polyclinics/PHPCs) from 13 April 2020 to 31 December 2022 with ARI symptoms and tested negative for SARS-CoV-2. They were randomly assigned a T_0_ following the distribution of infection dates among cases, and those who died within 30 days or had their first SARS-CoV-2 infection within 300 days of assigned T_0_ were excluded. Individuals with missing demographic data were also excluded.

### Prespecified Outcomes

New-incident cardiovascular, cerebrovascular, and other thrombotic complications in the postacute phase of COVID-19 were assessed during a follow-up period beginning 31 days post-T_0_ and ending 300 days post-T_0_. Diagnoses were based on *International Classification of Diseases, Tenth Revision* codes recorded in the Singapore national healthcare claims database, with reference to previous work on postacute sequelae of COVID-19 [5, 11, 22–24]. Cerebrovascular complications included stroke and transient ischemic attack (TIA). Cardiovascular complications included the following: dysrhythmias, including atrial fibrillation/flutter, tachycardia, bradycardia, and other arrhythmias; ischemic heart disease (IHD), including acute coronary disease, myocardial infarction, ischemic cardiomyopathy, and angina; other cardiac disorders, including heart failure, nonischemic cardiomyopathy, cardiac arrest, and cardiogenic shock; and inflammatory heart disease, including pericarditis and myocarditis. Thrombotic complications included pulmonary embolism, deep venous thrombosis, superficial venous thrombosis, and arterial thromboses. We defined 2 additional composite outcomes: MACE, which included the first incidence of myocardial infarction, stroke, heart failure, ventricular arrhythmia, or sudden cardiac death; and the composite of any new-incident cardiovascular, cerebrovascular, or other thrombotic complication.

### Statistical Analysis

We estimated the risks and excess burdens of prespecified new-incident cardiovascular, cerebrovascular, and other thrombotic complications for the SARS-CoV-2–infected group compared with uninfected test-negative controls, at 300 days from T_0_. For estimation of risks for each new-incident complication, a subcohort of individuals without history of the complication being reported was constructed. Individuals were excluded from each subcohort if they had reported in the past 5 years the specific complication being studied. Baseline characteristics of the SARS-CoV-2–infected and test-negative control groups, along with standardized mean difference (SMD) between groups, were computed.

We then adjusted for differences in baseline characteristics between comparison groups through inverse probability weighting, incorporating all available covariates: demographic characteristics (age, sex, ethnicity), socioeconomic status (housing type), vaccination status (not vaccinated, vaccinated, vaccinated and boosted), and comorbidity burden as indicated by constituent conditions within the Charlson comorbidity index. Although Charlson comorbidity index contained cardiovascular and cerebrovascular conditions including myocardial infarction, heart failure, stroke, and TIA, there was no overlap with subsequent outcome variables as individuals with preexisting cardiovascular/cerebrovascular conditions were already excluded at the subcohort construction stage. Housing type is a key marker of socioeconomic status in urbanized Singapore, where the majority (≥90%) stay in public housing under a tiered subsidy scheme; smaller-sized flats are more heavily subsidized and have caps on eligibility based on household income [25].

In each subcohort, a propensity score of belonging to the infected group was computed using a logistic regression that used the abovementioned covariates as explanatory terms. Inverse probability weights were computed as 1 / propensity score for cases; and 1 / (1 – propensity score) for test-negative controls. SMDs were used to assess covariate balance after inverse probability weighting.

Hazard ratios (HRs) of incident complications between the SARS-CoV-2–infected and test-negative control groups were then estimated using cause-specific hazard models with death taken as a competing risk, with inverse probability weights applied. A doubly robust approach was employed for each model, where covariates used to construct inverse probability weights were included in each model specification as explanatory variables. This approach was used to prevent model misspecification in the generation of inverse probability weights or HRs in subsequent analyses.

Burden per 1000 individuals at 300 days of follow-up and the excess burden of cardiovascular, cerebrovascular, and other thrombotic complications were estimated on the basis of the difference between the estimated incidence rates in both groups. Excess burdens here were defined as the increase/decrease in incidence rate in SARS-CoV-2–infected individuals versus test-negative individuals.

Previous studies have shown a dose-response relationship between increased severity of initial infection, older age, female sex, and full vaccination with postacute sequelae of SARS-CoV-2 infection [26]. We therefore also examined effect modification, by conducting analyses in subgroups by age (18–65 years, ≥66 years), ethnicity (Chinese, Malay, Indian, or other), sex (male or female), socioeconomic status, acute-phase disease severity (mild, hospitalized, or severe), and vaccination status (unvaccinated, fully vaccinated, or fully vaccinated and boosted).

As part of sensitivity analyses, we subjected our analyses to a set of negative-outcome controls where no prior knowledge supports the existence of causal associations between SARS-CoV-2 exposure and the risks of negative-outcome controls. Specifically, we examined the risk of atopic dermatitis and various neoplasms (B-cell lymphoma, Hodgkin lymphoma, malignancy of tongue) post-SARS-CoV-2 infection as negative-outcome controls, in line with previously published approaches [5, 24], modified to remove conditions (eg. sickle-cell trait, melanoma) uncommon in our predominantly Asian population.

Estimation of variance when weightings were applied was accomplished using robust sandwich variance estimators, which were previously demonstrated to reproduce valid model estimates even under model misspecification [27]. A 95% confidence interval (CI) that excluded unity was considered evidence of statistical significance. Analyses were conducted using Stata version 16 software, and results were visualized using R version 4.04 software.

### Ethics Statement

This study was done as part of national public health research under the Infectious Diseases Act, Singapore; ethics review by an institutional review board was not required.

## RESULTS

### Baseline Characteristics

We included 1 684 085 test-negative controls and 106 012 cases with documented SARS-CoV-2 infection in our study. Demographic and clinical characteristics of both SARS-CoV-2–infected and test-negative control groups before and after propensity score matching is presented in Table 1. The mean age of participants in the SARS-CoV-2–infected group was 51 years with 80.7% and 10.8% fully vaccinated and boosted, respectively. Around 55.8% were male and 68.2% of Chinese ethnicity (Table 1). After weighting, differences in baseline characteristics between the 2 groups were small (Table 1).

**Table 1.** Baseline and Inverse Probability–Weighted Characteristics of the Study Population

Characteristic	Cases (n = 106 012)^a^	Controls (n = 1 684 085)^a^	Cases, Weighted	Controls, Weighted	Weighted SMD^b^
Age, y, mean (SD)	51 (17.25)	48 (17.7)	48 (17.0)	48 (17.7)	0.003
Vaccination					
Unvaccinated	9107 (8.59)	113 145 (6.7)	122 396 (6.8)	122 246 (6.83)	0.000
Fully vaccinated	85 500 (80.6)	1 268 223 (75.3)	1 345 416 (75.1)	1 353 727 (75.6)	0.012
Boosted	11 405 (10.8)	302 717 (18.0)	323 097 (18.0)	314 139 (17.6)	0.013
Ethnicity					
Chinese	72 252 (68.2)	1 274 509 (75.7)	134 7247 (75.2)	1 346 724 (75.2)	0.000
Malay	18 971 (17.9)	197 121 (11.7)	223 058 (12.5)	216 162 (12.1)	0.012
Indian	11 965 (11.3)	156 435 (9.29)	163 884 (9.2)	168 385 (9.41)	0.009
Other	2824 (2.7)	56 020 (3.33)	56 719 (3.2)	58 841 (3.29)	0.007
Sex					
Male	59 161 (55.8)	810 035 (48.1)	872 765 (48.7)	869 203 (48.6)	0.004
Housing type					
1–2 rooms	8513 (8.03)	71 102 (4.2)	79 711 (4.5)	79 622 (4.45)	0.000
3 rooms	24 684 (23.3)	269 371 (16.0)	294 540 (16.5)	294 069 (16.4)	0.001
4 rooms	37 492 (35.4)	533 771 (31.7)	572 116 (32.0)	571 263 (31.9)	0.001
5 rooms/Executive condominium	25 076 (23.7)	424 788 (25.2)	448 732 (25.1)	449 859 (25.1)	0.002
Private housing	10 247 (9.7)	385 053 (22.9)	395 809 (22.1)	395 299 (22.1)	0.000
Comorbidities					
Cerebrovascular disorders	341 (0.32)	3997 (0.24)	4383 (0.24)	4351 (0.24)	0.000
Dysrhythmias	413 (0.39)	3871 (0.23)	5433 (0.30)	4193 (0.23)	0.013
Ischemic heart disease	540 (0.51)	5604 (0.33)	6424 (0.36)	6107 (0.34)	0.003
Other cardiac disorders	320 (0.30)	2585 (0.15)	3310 (0.18)	2850 (0.16)	0.006
Thrombotic disorders	98 (0.09)	920 (0.05)	1201 (0.07)	1003 (0.06)	0.004
Inflammatory heart disease	3 (0.00)	24 (0.00)	41 (>0.00)	25 (>0.00)	0.002

Data are presented as No. (%) unless otherwise indicated.

Abbreviations: COVID-19, coronavirus disease 2019; SD, standard deviation; SMD, standardized mean difference.

^a^Number of cases and controls after inclusion and exclusion criteria were met.

^b^SMD after inverse probability weighting of cases (COVID-19–infected individuals) and controls (uninfected individuals).

We estimated the HRs of prespecified cardiovascular, cerebrovascular, and other thrombotic complications in the SARS-CoV-2–infected and test-negative control groups (Table 2).

**Table 2.** Hazard Ratios and Excess Burdens of Prespecified Complications in the Coronavirus Disease 2019–Exposed Group and Control Groups

Outcome	HR (95% CI)^a^	Excess Burden (95% CI) (Weighted, per 1000 Persons)	No. of Individuals			
			Controls	Controls With Outcome	Cases	Cases With Outcome
Composite outcomes						
Any cardiovascular, cerebrovascular, and other thrombotic complication	1.16 (1.07–1.25)^b^	0.70 (.53–.88)^b^	1 607 157	9970	99 214	912
Major adverse cardiovascular/cerebrovascular events	1.14 (1.02–1.26)^b^	0.28 (.15–.40)^b^	1 656 164	5657	103 215	560
Cerebrovascular complications (eg, stroke, TIA)	1.12 (.973–1.29)	0.15 (.05–.24)^b^	1 655 044	3276	103 439	290
Cardiovascular complications						
Dysrhythmias	1.32 (1.15–1.52)^b^	0.51 (.42–.61)^b^	1 664 562	3071	104 408	311
Ischemic heart diseases	1.04 (.92–1.17)	0.02 (−.08 to .13)	1 652 224	4391	103 083	392
Other cardiac disorders	1.33 (1.14–1.55)^b^	0.30 (.22–.37)^b^	1 676 187	1895	105 132	236
Inflammatory heart disease	1.76 (.47–6.64)	0.01 (.00–.02)	1 683 885	23	105 994	3
Other thrombotic complications (eg. pulmonary embolism, venous thromboses, arterial thromboses)	1.22 (.94–1.58)	0.08 (.04–.13)^b^	1 680 478	809	105 662	84
Individual outcomes						
Stroke	1.06 (.91–1.23)	0.02 (−.07 to .10)	1 658 327	2776	103 688	243
TIA	1.21 (.90–1.64)	0.08 (.04–.13)^b^	1 678 949	733	105 584	63
Atrial fibrillation	1.15 (.94–1.40)	0.06 (−.00 to .13)	1 675 638	1521	105 259	142
Sinus tachycardia	1.03 (.76–1.40)	0.01 (−.03 to .05)	1 680 493	652	105 716	58
Sinus bradycardia	1.64 (1.12–2.41)^b^	0.10 (.07–.13)^b^	1 682 277	272	105 859	39
Other arrhythmia	1.68 (1.34–2.12)^b^	0.39 (.33–.45)^b^	1 676 468	1006	105 420	118
Myocardial infarction	1.08 (.92–1.27)	0.06 (−.03 to .14)	1 671 974	2454	104 773	235
Acute coronary disease	0.96 (.83–1.11)	−0.11 (−.19 to −.02)	1 661 961	2882	104 036	238
Ischemic cardiomyopathy	1.11 (.67–1.85)	0.01 (−.01 to .04)	1 682 982	228	105 866	21
Angina	1.24 (.96–1.61)	0.11 (.06–.15)^b^	1 673 700	802	105 038	87
Heart failure	1.28 (1.08–1.52)^b^	0.18 (.11–.25)^b^	1 677 494	1524	105 270	188
Nonischemic cardiomyopathy	1.63 (1.14–2.32)^b^	0.11 (.08–.14)^b^	1 682 194	297	105 813	43
Cardiac arrest	1.17 (.68–2.02)	0.01 (−.01 to .03)	1 683 998	188	106 000	19
Cardiogenic shock	0.76 (.36–1.60)	−0.01 (−.03 to −.00)	1 683 937	81	106 000	8
Pulmonary embolism	1.13 (.68–1.85)	0.01 (−.02 to .04)	1 683 139	257	105 932	21
Deep venous thrombosis	1.12 (.82–1.54)	0.03 (−.01 to .06)	1 681 727	509	105 767	56
Superficial venous thromboses	1.78 (.83–3.84)	0.04 (.02–.06)^b^	1 683 640	88	105 970	10
Arterial thromboses	0.82 (.23–2.90)	−0.01 (−.02 to .00)	1 683 809	47	105 990	3
Pericarditis	…^c^	0.02 (.01–.02)^b^	1 683 951	13	105 998	3
Myocarditis	…^c^	−0.01 (−.01 to −.00)	1 684 013	12	106 008	0

HR >1 denotes higher risk of a respective composite/individual new cardiovascular, cerebrovascular, and other thrombotic complications in the coronavirus disease 2019 (COVID-19)–exposed group versus control group. Excess burden >0 denotes excess burden in a respective composite/individual new cardiovascular, cerebrovascular, and other thrombotic complications in the COVID-19–exposed group versus control group.

Abbreviations: CI, confidence interval; HR, hazard ratio; TIA, transient ischemic attack.

^a^Each model is inverse probability weighted and regression adjusted based on demographic characteristics (age, sex, ethnicity), socioeconomic status (housing type), vaccination status (not vaccinated, vaccinated, vaccinated and boosted), and comorbidity burden at baseline (constituent conditions in Charlson comorbidity index).

^b^Denotes 95% CIs that are bounded away from 0.

^c^Not estimable due to 0 myocarditis cases in the COVID-19–exposed group.

Among COVID-19 survivors, there were increased risks of both composite endpoints, namely MACE (HR, 1.14 [95% CI, 1.02–1.26]) and any cardiovascular, cerebrovascular, or other thrombotic complication (HR, 1.16 [95% CI, 1.07–1.25]) compared with the control group.

COVID-19 survivors did not exhibit higher risks of all cerebrovascular complications (HR, 1.12 [95% CI, .97–1.29]), such as stroke (HR, 1.056 [95% CI, .908–1.23]) and TIA (HR, 1.21 [95% CI, .90–1.64]), although there was a moderate weighted excess burden (EB) per 1000 persons in the COVID-19–exposed group for TIA (EB, 0.08 [95% CI, .04–.13]).

With respect to cardiovascular complications, there were increased risks and excess burdens of dysrhythmias (HR, 1.32 [95% CI, 1.15–1.52]), specifically, sinus bradycardia (HR, 1.64 [95% CI, 1.12–2.41]) and other arrhythmias (HR, 1.68 [95% CI, 1.34–2.12]), among the COVID-19 survivors. COVID-19 survivors did not exhibit higher risk of all inflammatory heart disease (HR, 1.76 [95% CI, .47–6.64]).

SARS-CoV-2 infection was not positively associated with all IHD (HR, 1.04 [95% CI, .92–1.17]). There was no increased risk of acute coronary disease (HR, 0.960 [95% CI, .83–1.11]), myocardial infarction (HR, 1.08 [95% CI, .92–1.27]) ischemic cardiomyopathy (HR, 1.11 [95% CI, .67–1.85]), or angina (HR, 1.24 [95% CI, .96–1.61]) in COVID-19 survivors. However, there was a moderate weighted excess burden per 1000 persons in the COVID-19 group for angina (EB, 0.11 [95% CI, .06–.15]).

There were increased risks of other cardiac disorders (HR, 1.33 [95% CI, 1.14–1.55]), namely, heart failure (HR, 1.28 [95% CI, 1.08–1.52]) and nonischemic cardiomyopathy (HR, 1.63 [95% CI, 1.14–2.32]).

Risk of other thrombotic complications (HR, 1.22 [95% CI, .94–1.58]), including pulmonary embolism (HR, 1.13 [95% CI, .68–1.85]), deep venous thrombosis (HR, 1.12 [95% CI, .815–1.54]), superficial vein thrombosis (HR, 1.78 [95% CI, .83–3.84]), and arterial thromboses (HR, 0.816 [95% CI, .230–2.90]), were not increased in COVID-19 survivors.

### Subgroup Analysis

**Figure 2.**
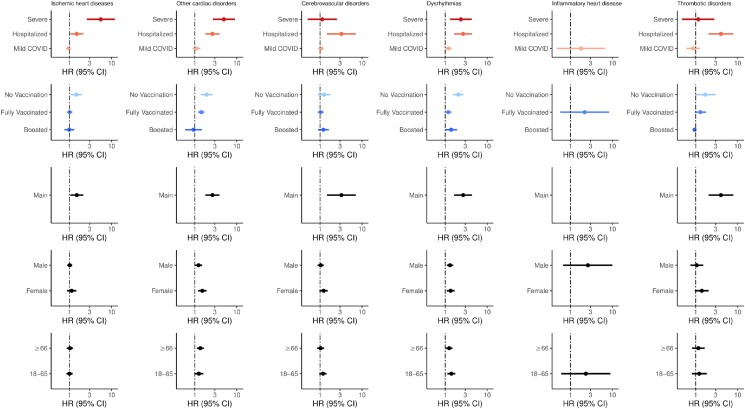
Hazard ratios (HRs) and 95% confidence intervals (CIs) for composite cardiovascular, cerebrovascular, and other thrombotic complications according to severity, vaccination, main cohort, sex, and age group (in years). Points and lines represent point estimates for HRs and 95% CIs, respectively. Blank points represent inestimable HRs due to null counts in that specific subgroup for cases, controls, or both. Abbreviations: CI, confidence interval; COVID, coronavirus disease 2019; HR, hazard ratio.

We examined the risk and excess burdens of new-incident complications in subgroups (Figure 2) based on vaccination status (unvaccinated, vaccinated, vaccinated and boosted), severity of acute infection (mild, hospitalized, severe), sex (male, female), age (18–65, ≥66 years), and socioeconomic status (by housing type). Full model estimates are provided in the Supplemental Tables 2–20.

Among nonhospitalized COVID-19 survivors, there was no increased risk of cardiovascular, cerebrovascular, and other thrombotic complications (Figure 2) except for dysrhythmias (HR, 1.20 [95% CI, 1.02–1.42]). However, risks of complications were increased in the hospitalized group, including cerebrovascular complications (HR, 3.23 [95% CI, 1.47–7.11]), dysrhythmias (HR, 2.67 [95% CI, 1.64–4.36]), IHD (HR, 1.487 [95% CI, 1.05–2.11]), other cardiac disorders (HR, 2.63 [95% CI, 1.79–3.87]), and other thrombotic complications (HR, 4.01 [95% CI, 2.03–7.91]).

Compared with the control group, we found that unvaccinated COVID-19 survivors had higher risk of various cardiovascular complications, including IHD (HR, 1.45 [95% CI, 1.07–1.95]), dysrhythmias (HR, 2.04 [95% CI, 1.53–2.72]), and other cardiac disorders (HR, 1.92 [95% CI, 1.39–2.65]). These effect sizes were also larger compared with the main analysis (Figure 2, Table 3). There was also increased risk for composite outcomes, including any cardiovascular, cerebrovascular, or other thrombotic complication (HR, 1.56 [95% CI, 1.29–1.90]) and MACE (HR, 1.51 [95% CI, 1.19–1.92]). However, vaccination and subsequent boosting decreased risk of new-incident complications associated in a graded fashion. Specifically, all HRs for the vaccinated COVID-19 survivors were smaller compared with estimates in the main analysis (Figure 2, Table 3). Overall, there were no increased risks of any cardiovascular, cerebrovascular, or other thrombotic complication (HR, 1.10 [95% CI, .92–1.32]) among boosted COVID-19 survivors. No HRs for individual complications were significant in the boosted group, except for other arrhythmia (HR, 2.20 [95% CI, 1.40–3.46]).

**Table 3.** Hazard Ratios and Excess Burdens of Prespecified Cardiovascular, Cerebrovascular, or Other Thrombotic Complications in the Coronavirus Disease 2019 and Control Groups for Each Vaccination Subgroup

Outcome	Unvaccinated		Fully Vaccinated		Boosted	
	HR (95% CI)^a^	Excess Burden (95% CI) (Weighted, per 1000 Persons)	HR (95% CI)^a^	Excess Burden (95% CI) (Weighted, per 1000 Persons)	HR (95% CI)^a^	Excess Burden (95% CI) (Weighted, per 1000 Persons)
Composite outcomes						
Any cardiovascular, cerebrovascular, and other thrombotic complication	1.56 (1.29–1.90)^b^	3.95 (3.14–4.75)	1.11 (1.02–1.22)^b^	0.41 (.24–.59)	1.10 (.92–1.32)	1.11 (.55–1.68)
Major adverse cardiovascular/cerebrovascular events	1.51 (1.19–1.92)^b^	2.13 (1.51–2.75)	1.12 (.99–1.26)	0.20 (.07–.33)	1.05 (.81–1.35)	0.27 (−.13 to .68)
Cerebrovascular complications (eg. stroke, TIA)	1.25 (.89–1.76)	0.61 (.16–1.05)	1.04 (.88–1.22)	0.01 (−.09 to .10)	1.20 (.89–1.62)	0.75 (.43–1.07)
Cardiovascular complications						
Dysrhythmias	2.04 (1.53–2.72)^b^	2.58 (2.07–3.08)	1.18 (1.00–1.40)^b^	0.24 (.14–.33)	1.38 (1.00–1.91)^b^	1.12 (.82–1.42)
Ischemic heart diseases	1.45 (1.07–1.95)^b^	1.22 (.72–1.71)	1.00 (.87–1.16)	−0.03 (−.14 to .08)	0.98 (.75–1.29)	−0.10 (−.46 to .26)
Other cardiac disorders	1.92 (1.39–2.65)^b^	1.60 (1.19–2.02)	1.43 (1.19–1.70)^b^	0.31 (.24–.39)	0.93 (.59–1.46)	−0.16 (−.38 to .06)
Inflammatory heart disease	…^c^	0.00 (.00–.00)	2.18 (.57–8.36)	0.02 (.00–.03)	…^c^	−0.01 (−.03 to −.00)
Other thrombotic complications (eg. pulmonary embolism, venous thromboses, arterial thromboses)	1.68 (.95–2.95)	0.50 (.24–.76)	1.30 (.95–1.79)	0.11 (.05–.16)	0.94 (.42–2.12)	−0.10 (−.22 to .03)
Individual outcomes						
Stroke	1.27 (.89–1.82)	0.61 (.18–1.03)	0.98 (.82–1.17)	−0.07 (−.16 to .01)	1.13 (.80–1.59)	0.39 (.10–.68)
TIA	0.74 (.23–2.38)	−0.14 (−.30 to .02)	1.20 (.83–1.73)	0.06 (.02–.11)	1.25 (.72–2.19)	0.21 (.06–.37)
Atrial fibrillation	1.65 (1.11–2.46)^b^	0.84 (.50–1.18)	1.14 (.90–1.44)	0.05 (−.01 to .11)	1.07 (.64–1.78)	0.10 (−.11 to .31)
Sinus tachycardia	2.59 (1.39–4.81)^b^	0.92 (.66–1.18)	0.82 (.56–1.19)	−0.07 (−.11 to −.03)	0.95 (.40–2.26)	−0.03 (−.14 to .08)
Sinus bradycardia	4.58 (1.49–14.07)^b^	0.42 (.27–.58)	1.40 (.87–2.25)	0.05 (.02–.08)	1.39 (.55–3.50)	0.10 (.01–.18)
Other arrhythmia	2.15 (1.29–3.60)^b^	0.89 (.61–1.17)	1.40 (1.05–1.86)^b^	0.20 (.14–.26)	2.20 (1.40–3.46)^b^	1.13 (.94–1.32)
Myocardial infarction	1.42 (.99–2.03)	0.68 (.29–1.06)	0.66 (.35–1.24)	−0.11 (−.19 to −.03)	1.25 (.88–1.77)	0.64 (.37–.91)
Acute coronary disease	1.11 (.69–1.77)	0.19 (−.14 to .52)	0.97 (.82–1.15)	−0.05 (−.14 to .03)	0.86 (.63–1.19)	−0.46 (−.75 to −.18)
Ischemic cardiomyopathy	1.34 (.52–3.46)	0.09 (−.06 to .24)	0.81 (.39–1.66)	−0.02 (−.04 to .01)	1.65 (.63–4.32)	0.13 (.04–.22)
Angina	2.17 (1.09–4.32)^b^	0.46 (.26–.66)	1.32 (.97–1.79)	0.12 (.07–.17)	0.82 (.44–1.51)	−0.15 (−.29 to −.01)
Heart failure	2.04 (1.44–2.90)^b^	1.48 (1.10–1.86)	1.45 (1.19–1.77)^b^	0.25 (.19–.32)	0.70 (.39–1.26)	−0.51 (−.70 to −.32)
Nonischemic cardiomyopathy	1.67 (.72–3.83)	0.17 (.02–.32)	1.48 (.98–2.23)	0.07 (.04–.10)	1.79 (.82–3.93)	0.23 (.13–.33)
Cardiac arrest	1.01 (.29–3.52)	0.00 (−.14 to .14)	0.75 (.10–5.95)	0.00 (−.02 to .02)	1.58 (.45–5.63)	0.10 (.03–.18)
Cardiogenic shock	0.61 (.22–1.68)	−0.02 (−.07 to .04)	1.01 (.45–2.27)	−0.00 (−.02 to .01)	…^c^	−0.07 (−.10 to −.04)
Pericarditis	…^c^	0.00 (.00–.00)	…^c^	0.02 (.01–.03)	…^c^	−0.01 (−.02 to .00)
Myocarditis	…^c^	0.00 (.00–.00)	…^c^	−0.01 (−.01 to −.00)	…^c^	−0.01 (−.02 to .00)
Pulmonary embolism	1.52 (.46–5.06)	0.13 (−.00 to .27)	0.83 (.45–1.52)	−0.03 (−.05 to −.00)	1.91 (.67–5.47)	0.17 (.08–.26)
Deep venous thrombosis	1.85 (.95–3.60)	0.36 (.15–.56)	1.27 (.87–1.86)	0.06 (.02–.10)	0.47 (.14–1.60)	−0.25 (−.34 to −.16)
Superficial venous thromboses	1.47 (.21–10.35)	0.03 (−.04 to .10)	2.57 (1.13–5.87)^b^	0.07 (.05–.09)	…^c^	−0.08 (−.11 to −.05)
Arterial thromboses	1.30 (.45–3.75)	0.05 (−.03 to .13)	0.20 (.03–1.53)	−0.02 (−.03 to −.01)	0.93 (.51–1.69)	0.03 (−.01 to .07)

HR >1 denotes higher risk of a respective composite/individual new cardiovascular, cerebrovascular, or other thrombotic complication in the coronavirus disease 2019 (COVID-19)–exposed group versus control group. Excess burden >0 denotes excess burden in a respective composite/individual new cardiovascular, cerebrovascular, or other thrombotic complication in the COVID-19–exposed group versus control group.

Abbreviations: CI, confidence interval; HR, hazard ratio; TIA, transient ischemic attack.

^a^Each model is inverse probability weighted and regression adjusted based on demographic characteristics (age, sex, ethnicity), socioeconomic status (housing type), vaccination status (not vaccinated, vaccinated, vaccinated and boosted), and comorbidity burden at baseline (constituent conditions in Charlson comorbidity index).

^b^Denotes 95% CIs that are bounded away from 0.

^c^Not estimable due to 0 cases in either group.

Subgroup analysis based on sex and age did not change HR estimates drastically (Figure 2; Supplementary Tables 2–5).

### Sensitivity Analyses

For conditions included as negative-outcome controls, such as B-cell lymphoma, malignancy of tongue, and atopic dermatitis, all HRs had CIs that were found to cross the zero bound (Supplementary Table 20).

## DISCUSSION

In our large retrospective population-wide cohort study involving 2 912 069 test-negative controls and 111 799 cases infected during transmission of the Delta variant, infected individuals had higher risks of specific cardiovascular complications, including dysrhythmias and other cardiac disorders—such as heart failure and nonischemic cardiomyopathy—in the 300-day follow-up period postinfection. Chronic inflammation arising from residual viral reservoirs persisting in cardiac tissue or an autoimmune response to cardiac antigens may result in tissue damage and myocardial fibrosis, leading to impaired ventricular contractility and potential reentrant arrhythmias [2, 3]. Virus-induced lung injury may also induce right ventricular dysfunction through increasing pulmonary vascular resistance and right ventricular strain [28]. Prospective observational studies have documented persistent rate and rhythm abnormalities even after relatively mild infection [29, 30] and, similarly, reductions in left ventricular ejection fraction, right ventricular function, and diastolic dysfunction 3–9 months after acute COVID-19 [30, 31]. Further studies are needed to clarify the potential pathophysiological mechanism underlying these associations.

In our multiethnic Southeast Asian population, we reported 1.45–2.04 times higher risk of various cardiovascular complications, including IHD, dysrhythmias, and other cardiac disorders in the unvaccinated, infected group. These results are consistent with Xie et al, who reported approximately 1.5–2.0 times higher risk of various cardiovascular complications postinfection among US Veterans [5]. However, we did not observe elevated thrombotic risk, in contrast to other studies demonstrating increased risk of thrombotic complications in the United States (US) and United Kingdom [5–11]. Asian populations may have different thrombogenic phenotypes [32]. In the acute phase of COVID-19, <2% of a multicenter Japanese cohort had thrombotic complications [33], compared with rates of 7%–14% reported from a pooled meta-analysis of hospitalized studies conducted in Western populations [34]. Additional studies evaluating post-COVID-19 complications in other Asian populations are warranted.

In our study, the risk of cardiovascular complications postacute infection was evident even in mild cases who were not hospitalized. Large population-based studies in the US have demonstrated a similar dose-response relationship with increasing severity [5, 8, 34]. Risk was increasingly attenuated if individuals were vaccinated/boosted; similarly, Al-Aly et al reported lower risk of new-incident cardiovascular and cerebrovascular complications in vaccinated US Veterans with breakthrough SARS-CoV-2 infection compared with unvaccinated cases [11]. We did not observe significantly increased risk of individual cardiovascular, cerebrovascular, or other thrombotic complications in the boosted group, except for other arrhythmias. Electrocardiographic and cardiac conduction abnormalities are not uncommonly reported among unvaccinated patients on long-term follow-up with moderate to severe COVID-19; in a Chinese cohort, 16.3% reported arrhythmias at 12 months postdischarge [35]. While periodic boosters are required as part of prevention strategies to ameliorate postacute sequelae, this alone may not fully mitigate risks, given waning [36] and possible vaccine escape with new variants [37]. While the excess burdens of post-COVID-19 cardiovascular and cerebrovascular complications seem small (<10 per 1000 person-), the long-term burden of cardiovascular complications may be substantial. Updates to vaccination, such as bivalent vaccines, and early treatment with therapeutics [38] will potentially play a key role in mitigating the risks of long-term sequelae of post-SARS-CoV-2 infection.

Our study has the following limitations. The control group might be contaminated by undiagnosed or asymptomatic SARS-CoV-2 infections, causing differential misclassification bias. Misclassification would bias estimated HRs and excess burdens downward, resulting in more conservative estimates. However, we utilized a comprehensive nationwide testing database during a period when diagnostic tests were widely available in primary care, testing was free and strongly encouraged, and reporting was mandatory [18]. Our cohort comprised a predominantly Chinese (75.23%) population, which may limit generalizability. While we used a large, comprehensive national healthcare claims database to examine a prespecified, comprehensive list of complications, we cannot rule out unobserved confounding. We did not adjust for several important comorbidities (hypertension and obesity) as physiological measurements (eg, blood pressure, body mass index) were not recorded in electronic health records. Survivor bias may also result in underestimates of the HR reported for various outcomes.

## CONCLUSIONS

In a national cohort of Delta-infected individuals, increased risk and burden of new-incident cardiovascular/cerebrovascular complications was demonstrated 300 days postinfection. Risks increased in a graded fashion according to initial severity; vaccination and boosting significantly attenuated risk.

## Supplementary Data

Supplementary materials are available at *Clinical Infectious Diseases* online. Consisting of data provided by the authors to benefit the reader, the posted materials are not copyedited and are the sole responsibility of the authors, so questions or comments should be addressed to the corresponding author.

## Supplementary Material

ciad469_Supplementary_Data

## Notes

***Financial support*.** J. T. L. is supported by the Ministry of Education, Singapore (Start-up Grant).
