# Supplementary material for: Long-term Cardiovascular, Cerebrovascular, and Other Thrombotic Complications in COVID-19 Survivors: A Retrospective Cohort Study
Source: Clin Infect Dis. 2023 Sep 25;78(1):70–9. doi: 10.1093/cid/ciad469 (PMC10810710; doi:10.1093/cid/ciad469)
Supplement: ciad469_Supplementary_Data [file ciad469_supplementary_data.docx]

Table of Contents

[Table 1: List of ICD-10 codes used for outcomes of interest 4](#_Toc141475521)

[Plot 1: Hazards ratio (HR) and 95% CIs for individual cardiovascular, cerebrovascular or other thrombotic complications according to severity subgroups in the acute phase of COVID-19 infection 6](#_Toc141475522)

[Plot 2: excess burdens and 95% CIs for individual cardiovascular, cerebrovascular or other thrombotic complications according to severity subgroups in the acute phase of COVID-19 infection 7](#_Toc141475523)

[Plot 3: Hazards ratio (HR) and 95% CIs for individual cardiovascular, cerebrovascular or other thrombotic complications according to vaccination subgroups in the acute phase of COVID-19 infection 8](#_Toc141475524)

[Plot 4: Excess burdens per 1000 and 95% CIs for individual cardiovascular, cerebrovascular or other thrombotic complications according to vaccination subgroups in the acute phase of COVID-19 infection 9](#_Toc141475526)

[Table 3: Subgroup analysis: hazards ratios and excess burdens per 1000 person years for females 11](#_Toc141475527)

[Table 4: Subgroup analysis: hazards ratios and excess burdens per 1000 person years for age 18 – 65 12](#_Toc141475528)

[Table 5: Subgroup analysis: hazards ratios and excess burdens per 1000 person years for age 66+ 13](#_Toc141475529)

[Table 6: Subgroup analysis: hazards ratios and excess burdens per 1000 person years for unvaccinated 14](#_Toc141475530)

[Table 7: Subgroup analysis: hazards ratios and excess burdens per 1000 person years for fully vaccinated 15](#_Toc141475531)

[Table 8: Subgroup analysis: hazards ratios and excess burdens per 1000 person years for fully vaccinated and boosted 16](#_Toc141475532)

[Table 9: Subgroup analysis: hazards ratios and excess burdens per 1000 person years for Chinese 17](#_Toc141475533)

[Table 10: Subgroup analysis: hazards ratios and excess burdens per 1000 person years for Malay 18](#_Toc141475534)

[Table 11: Subgroup analysis: hazards ratios and excess burdens per 1000 person years for Indian 19](#_Toc141475535)

[Table 12: Subgroup analysis: hazards ratios and excess burdens per 1000 person years for Other Ethnicities 20](#_Toc141475536)

[Table 13: Main analysis with inverse probability weights without covariates in regression specification: hazards ratios and excess burdens per 1000 person years 21](#_Toc141475537)

[Table 14: Subgroup analysis with inverse probability weights without covariates in regression specification: hazards ratios and excess burdens per 1000 person years for unvaccinated 22](#_Toc141475538)

[Table 15: Subgroup analysis with inverse probability weights without covariates in regression specification: hazards ratios and excess burdens per 1000 person years for fully vaccinated 23](#_Toc141475539)

[Table 16: Subgroup analysis with inverse probability weights without covariates in regression specification: hazards ratios and excess burdens per 1000 person years for fully vaccinated and boosted 24](#_Toc141475540)

[Table 17: Subgroup analysis with inverse probability weights without covariates in regression specification: hazards ratios and excess burdens per 1000 person years for severe cases 25](#_Toc141475541)

[Table 18: Subgroup analysis with inverse probability weights without covariates in regression specification: hazards ratios and excess burdens per 1000 person years for hospitalised cases 26](#_Toc141475542)

[Table 19: Subgroup analysis with inverse probability weights without covariates in regression specification: hazards ratios and excess burdens per 1000 person years for mild cases 27](#_Toc141475543)

[Table 20: Negative outcome controls: hazards ratios and excess burdens per 1000 person years 28](#_Toc141475544)

# Table 1: List of ICD-10 codes used for outcomes of interest

| **Outcome** | **ICD 10 Code** | **Description** |  |
| --- | --- | --- | --- |
| Stroke | I60 | Nontraumatic subarachnoid hemorrhage |  |
|  | I61 | Intracerebral hemorrhage |  |
|  | I62 | Other non-traumatic intracranial hemorrhage |  |
|  | I63 | Cerebral infarction |  |
|  | I64 | Stroke, unspecified (not specified as hemorrhage or infarction) |  |
|  | I67 | Other cerebrovascular diseases |  |
|  | I63.6 | Cerebral infarction due to cerebral venous thrombosis, nonpyogenic |  |
| Transient ischemic attack (TIA) | I65 | Occlusion and stenosis of precerebral arteries not resulting in cerebral infarction |  |
|  | I66 | Occlusion and stenosis of cerebral arteries not resulting in cerebral infarction |  |
|  | G45 | Transient cerebral ischaemic attacks and related syndromes |  |
|  | G46 | Vascular syndromes of brain in cerebrovascular diseases |  |
| Atrial fibrillation | I48 | Atrial fibrillation and flutter |  |
| Sinus tachycardia | I47 | Paroxysmal tachycardia |  |
|  | R00.0 | Tachycardia, unspecified |  |
| Sinus bradycardia | R00.1 | Bradycardia, unspecified |  |
| Other arrhythmias | I44 | Atrioventricular and left bundle-branch block |  |
|  | R00.2 | Palpitations |  |
|  | R00.8 | Other abnormalities of heart beat |  |
|  | R00.9 | Unspecified abnormalities of heart beat |  |
|  | I45 | Other conduction disorders |  |
|  | I49 | Other cardiac arrhythmias |  |
| Pericarditis | I30 | Acute pericarditis |  |
|  | B33.23 | Viral pericarditis |  |
| Myocarditis | B33.20 | Viral carditis, unspecified |  |
|  | B33.21 | Viral endocarditis |  |
|  | B33.22 | Viral myocarditis |  |
|  | B33.24 | Viral cardiomyopathy |  |
|  | I40 | Acute myocarditis |  |
| Myocardial infarction | I21 | Acute myocardial infarction |  |
|  | I22 | Subsequent ST elevation (STEMI) and non-ST elevation (NSTEMI) myocardial infarction |  |
| Acute coronary disease | I24 | Other acute ischemic heart diseases |  |
|  | I25.10 | Atherosclerotic heart disease of native coronary artery without angina pectoris |  |
|  | I25.11 | Atherosclerotic heart disease of native coronary artery with angina pectoris |  |
| Ischemic cardiomyopathy | I25.5 | Ischemic cardiomyopathy |  |
| Angina | I20 | Angina pectoris |  |
| Heart failure | I50 | Heart failure |  |
|  | I50.21 | Acute systolic (congestive) heart failure |  |
|  | 150.23 | Acute on chronic systolic (congestive) heart failure |  |
|  | 150.31 | Acute diastolic (congestive) heart failure |  |
|  | I50.33 | Acute on chronic diastolic (congestive) heart failure |  |
|  | I50.41 | Acute combined systolic (congestive) and diastolic (congestive) heart failure |  |
|  | I50.43 | Acute on chronic combined systolic (congestive) and diastolic (congestive) heart failure |  |
|  | I50.811 | Acute right heart failure |  |
|  | I50.813 | Acute on chronic right heart failure |  |
| Non-ischemic cardiomyopathy | I42 | Cardiomyopathy |  |
| Cardiac arrest | I46 | Cardiac arrest |  |
| Cardiogenic shock | R57.0 | Cardiogenic shock |  |
| Pulmonary embolism | I26 | Pulmonary embolism |  |
| Deep venous thrombosis | I80.1 | Phlebitis and thrombophlebitis of femoral vein |  |
|  | I80.2 | Phlebitis and thrombophlebitis of other and unspecified deep vessels of lower extremities |  |
|  | I80.8 | Phlebitis and thrombophlebitis of other sites |  |
|  | I81 | Portal vein thrombosis |  |
|  | I82 | Other venous embolism and thrombosis |  |
|  | I67.6 | Nonpyogenic thrombosis of intracranial venous system |  |
| Superficial venous thrombosis | I80.0 | Phlebitis and thrombophlebitis of superficial vessels of lower extremities |  |
|  | I80.3 | Phlebitis and thrombophlebitis of lower extremities, unspecified |  |
|  | I80.9 | Phlebitis and thrombophlebitis of unspecified site |  |
| Arterial thromboses | I74.0 | Embolism and thrombosis of abdominal aorta |  |
|  | I74.1 | Embolism and thrombosis of other and unspecified parts of aorta |  |
|  | I74.2 | Embolism and thrombosis of arteries of the upper extremities |  |
|  | I74.3 | Embolism and thrombosis of arteries of the lower extremities |  |
|  | I74.4 | Embolism and thrombosis of arteries of extremities, unspecified |  |
|  | I74.5 | Embolism and thrombosis of iliac artery |  |
|  | I74.8 | Embolism and thrombosis of other arteries |  |
|  | I74.9 | Embolism and thrombosis of unspecified artery |  |

# Plot 1: Hazards ratio (HR) and 95% CIs for individual cardiovascular, cerebrovascular or other thrombotic complications according to severity subgroups in the acute phase of COVID-19 infection

Hazards ratio (HR) and 95% CIs for individual cardiovascular, cerebrovascular or other thrombotic complications according to severity subgroups in the acute phase of COVID-19 infection. Points and lines represent point estimates for HRs and 95% CIs respectively. Blank points represent inestimable HRs due to null counts in that specific subgroup for cases, controls or both.

# Plot 2: excess burdens and 95% CIs for individual cardiovascular, cerebrovascular or other thrombotic complications according to severity subgroups in the acute phase of COVID-19 infection

Excess burdens (EB) per 1000 and 95% CIs for individual cardiovascular, cerebrovascular or other thrombotic complications according to severity subgroups in the acute phase of COVID-19 infection. Points and lines represent point estimates for HRs and 95% CIs respectively. Blank points represent inestimable HRs due to null counts in that specific subgroup for cases, controls or both.

# Plot 3: Hazards ratio (HR) and 95% CIs for individual cardiovascular, cerebrovascular or other thrombotic complications according to vaccination subgroups in the acute phase of COVID-19 infection

#

Hazards ratio (HR) and 95% CIs for individual cardiovascular, cerebrovascular or other thrombotic complications according to vaccination subgroups in the acute phase of COVID-19 infection. Points and lines represent point estimates for HRs and 95% CIs respectively. Blank points represent inestimable HRs due to null counts in that specific subgroup for cases, controls or both.

# Plot 4: Excess burdens per 1000 and 95% CIs for individual cardiovascular, cerebrovascular or other thrombotic complications according to vaccination subgroups in the acute phase of COVID-19 infection

Excess burdens (EB) per 1000 and 95% CIs for individual cardiovascular, cerebrovascular or other thrombotic complications according to severity subgroups in the acute phase of COVID-19 infection. Points and lines represent point estimates for HRs and 95% CIs respectively. Blank points represent inestimable HRs due to null counts in that specific subgroup for cases, controls or both.Table 2: Subgroup analysis: hazards ratios and excess burdens per 1000 person years for males

|  | No. of Individuals | | | |  |  |
| --- | --- | --- | --- | --- | --- | --- |
| Outcome | Controls | Controls with outcome | Cases | Cases with outcome | HR | Excess burden (weighted, per 1000 person years) |
| Any cardiovascular, cerebrovascular  and other thrombotic complications | 762917 | 5944 | 54607 | 580 | 1.09 (0.99, 1.20) | 0.43 (0.16, 0.71) |
| MACE | 791497 | 3564 | 57201 | 359 | 1.05 (0.92, 1.19) | 0.01 (-0.19, 0.21) |
| Cerebrovascular disorders | 793114 | 1862 | 57505 | 181 | 1.04 (0.87, 1.23) | 0.01 (-0.14, 0.16) |
| Dysrhythmias | 799904 | 1551 | 58228 | 180 | 1.30 (1.09, 1.55) | 0.51 (0.37, 0.65) |
| Ischemic heart diseases | 786774 | 3147 | 56928 | 293 | 1.01 (0.88, 1.16) | -0.07 (-0.26, 0.12) |
| Other cardiac disorders | 805477 | 1080 | 58618 | 138 | 1.23 (1.01, 1.50) | 0.25 (0.14, 0.37) |
| Thrombotic disorders | 808347 | 386 | 58952 | 44 | 1.06 (0.75, 1.51) | 0.02 (-0.05, 0.09) |
| Inflammatory heart disease | 809885 | 16 | 59146 | 3 | 2.60 (0.67, 10.0) | 0.03 (0.01, 0.05) |
| Stroke | 794959 | 1586 | 57663 | 152 | 1.00 (0.82,1.20) | -0.08 (-0.21, 0.05) |
| TIA | 807038 | 434 | 58881 | 39 | 1.04 (0.72, 1.49) | 0.00 (-0.07, 0.07) |
| Atrial fibrillation | 805292 | 794 | 58723 | 79 | 1.12 (0.86, 1.47) | 0.07 (-0.03, 0.16) |
| Sinus tachycardia | 808334 | 305 | 58985 | 34 | 1.15 (0.78, 1.69) | 0.05 (-0.01, 0.11) |
| Sinus bradycardia | 809090 | 128 | 59069 | 26 | 2.05 (1.29, 3.28) | 0.16 (0.12, 0.21) |
| Other arrhythmia | 806304 | 522 | 58827 | 65 | 1.42 (1.07, 1.88) | 0.25 (0.17, 0.34) |
| Myocardial infarction | 800834 | 1765 | 58206 | 175 | 1.03 (0.86, 1.24) | -0.01 (-0.15, 0.14) |
| Acute coronary disease | 793218 | 2190 | 57561 | 189 | 0.98 (0.83, 1.16) | -0.11 (-0.27, 0.04) |
| Ischemic cardiomyopathy | 809141 | 169 | 59034 | 17 | 0.98 (0.57, 1.71) | -0.01 (-0.05, 0.03) |
| Angina | 802661 | 561 | 58438 | 60 | 1.09 (0.80, 1.46) | 0.04 (-0.04, 0.12) |
| Heart failure | 806300 | 846 | 58712 | 102 | 1.10 (0.87, 1.39) | 0.06 (-0.04, 0.16) |
| Non ischemic cardiomyopathy | 808845 | 180 | 59031 | 31 | 1.88 (1.22, 2.90) | 0.19 (0.14, 0.25) |
| Cardiac arrest | 809970 | 119 | 59151 | 13 | 0.94 (0.51, 1.73) | -0.02 (-0.05, 0.02) |
| Cardiogenic shock | 809915 | 59 | 59150 | 7 | 0.87 (0.39, 1.93) | -0.01 (-0.04, 0.01) |
| Pulmonary embolism | 809632 | 123 | 59120 | 9 | 0.73 (0.35, 1.54) | -0.05 (-0.08, -0.01) |
| Deep venous thrombosis | 808900 | 244 | 59008 | 32 | 1.12 (0.75, 1.68) | 0.03 (-0.02, 0.09) |
| Superficial venous thromboses | 809829 | 41 | 59135 | 3 | 1.16 (0.32, 4.23) | 0.01 (-0.01, 0.03) |
| Arterial thromboses | 809872 | 23 | 59148 | 2 | 1.51 (0.45, 5.10) | 0.01 (-0.01, 0.03) |
| Pericarditis | 809927 | 11 | 59149 | 3 | 3.83 (0.95, 15.4) | 0.04 (0.02, 0.05) |
| Myocarditis | 809989 | 6 | 59158 | 0 | –^#^ | -0.01 (-0.01, -0.00) |

Hazards ratio (HR) and excess burdens of pre-specified cardiovascular complications in the COVID-19 and control groups. 95% confidence intervals are reported in parentheses.

*denotes 95% CIs which are bounded away from 0. ^#^not estimable due to 0 cases in either group.

HR > 1 denotes higher risk of a respective composite/individual new cardiovascular condition in the COVID-19 exposed group versus control group.

Excess burdens > 0 denotes excess burden in a respective composite/individual new cardiovascular condition in the COVID-19 exposed group versus control group

# Table 3: Subgroup analysis: hazards ratios and excess burdens per 1000 person years for females

|  | No. of Individuals | | | |  |  |
| --- | --- | --- | --- | --- | --- | --- |
| Outcome | Controls | Controls with outcome | Cases | Cases with outcome | HR | Excess burden (weighted, per 1000 person years) |
| Any cardiovascular, cerebrovascular  and other thrombotic complications | 844240 | 4026 | 44607 | 332 | 1.26 (1.11, 1.44) | 0.96 (0.74, 1.17) |
| MACE | 864667 | 2093 | 46014 | 201 | 1.32(1.11, 1.58) | 0.52 (0.37, 0.68) |
| Cerebrovascular disorders | 861930 | 1414 | 45934 | 109 | 1.22 (0.97, 1.53) | 0.28 (0.15, 0.40) |
| Dysrhythmias | 864658 | 1520 | 46180 | 131 | 1.34 (1.08, 1.67) | 0.54 (0.41, 0.67) |
| Ischemic heart diseases | 865450 | 1244 | 46155 | 99 | 1.12 (0.87, 1.45) | 0.08 (-0.04, 0.19) |
| Other cardiac disorders | 870710 | 815 | 46514 | 98 | 1.51 (1.19, 1.91) | 0.36 (0.26, 0.46) |
| Thrombotic disorders | 872131 | 423 | 46710 | 40 | 1.40 (0.95, 2.04) | 0.17 (0.10, 0.24) |
| Inflammatory heart disease | 874000 | 7 | 46848 | 0 | –^#^ | -0.01 (-0.01, -0.00) |
| Stroke | 863368 | 1190 | 46025 | 91 | 1.15(0.89,1.47) | 0.11 (-0.00, 0.22) |
| TIA | 871911 | 299 | 46703 | 24 | 1.47 (0.90, 2.39) | 0.15 (0.09, 0.21) |
| Atrial fibrillation | 870346 | 727 | 46536 | 63 | 1.20 (0.88, 1.64) | 0.09 (0.00, 0.17) |
| Sinus tachycardia | 872159 | 347 | 46731 | 24 | 0.91 (0.56, 1.48) | -0.04 (-0.10, 0.02) |
| Sinus bradycardia | 873187 | 144 | 46790 | 13 | 1.23 (0.64, 2.37) | 0.03 (-0.00, 0.07) |
| Other arrhythmia | 870164 | 484 | 46593 | 53 | 1.96 (1.38, 2.78) | 0.54 (0.45, 0.62) |
| Myocardial infarction | 871140 | 689 | 46567 | 60 | 1.23 (0.87, 1.74) | 0.11 (0.02, 0.19) |
| Acute coronary disease | 868743 | 692 | 46475 | 49 | 0.85 (0.62, 1.17) | -0.16 (-0.24, -0.08) |
| Ischemic cardiomyopathy | 873841 | 59 | 46832 | 4 | 1.64 (0.56, 4.75) | 0.03 (0.01, 0.06) |
| Angina | 871039 | 241 | 46600 | 27 | 1.59 (1.00, 2.53) | 0.15 (0.09, 0.20) |
| Heart failure | 871194 | 678 | 46558 | 86 | 1.55 (1.20, 1.99) | 0.32 (0.23, 0.41) |
| Non ischemic cardiomyopathy | 873349 | 117 | 46782 | 12 | 1.27 (0.65, 2.47) | 0.02 (-0.01, 0.06) |
| Cardiac arrest | 874028 | 69 | 46849 | 6 | 1.63 (0.62, 4.26) | 0.04 (0.01, 0.07) |
| Cardiogenic shock | 874022 | 22 | 46850 | 1 | 0.46 (0.06, 3.50) | -0.01 (-0.03, -0.00) |
| Pulmonary embolism | 873507 | 134 | 46812 | 12 | 1.53 (0.80, 2.91) | 0.08 (0.04, 0.12) |
| Deep venous thrombosis | 872827 | 265 | 46759 | 24 | 1.15 (0.70, 1.89) | 0.04 (-0.01, 0.09) |
| Superficial venous thromboses | 873811 | 47 | 46835 | 7 | 2.32 (0.90, 5.99) | 0.07 (0.04, 0.10) |
| Arterial thromboses | 873937 | 24 | 46842 | 1 | 0.23 (0.03, 1.73) | -0.02 (-0.03, -0.01) |
| Pericarditis | 874024 | 2 | 46849 | 0 | –^#^ | -0.00 (-0.01, 0.00) |
| Myocarditis | 874024 | 6 | 46850 | 0 | –^#^ | -0.01 (-0.01, -0.00) |

Hazards ratio (HR) and excess burdens of pre-specified cardiovascular complications in the COVID-19 and control groups. 95% confidence intervals are reported in parentheses.

*denotes 95% CIs which are bounded away from 0. ^#^not estimable due to 0 cases in either group.

HR > 1 denotes higher risk of a respective composite/individual new cardiovascular condition in the COVID-19 exposed group versus control group.

Excess burdens > 0 denotes excess burden in a respective composite/individual new cardiovascular condition in the COVID-19 exposed group versus control group

# Table 4: Subgroup analysis: hazards ratios and excess burdens per 1000 person years for age 18 – 65

|  | No. of Individuals | | | |  |  |
| --- | --- | --- | --- | --- | --- | --- |
| Outcome | Controls | Controls with outcome | Cases | Cases with outcome | HR | Excess burden (weighted, per 1000 person years) |
| Any cardiovascular, cerebrovascular  and other thrombotic complications | 1333122 | 4841 | 80962 | 448 | 1.13 (1.01, 1.27) | 0.44 (0.30, 0.59) |
| MACE | 1355333 | 2352 | 82777 | 221 | 1.03 (0.87, 1.21) | 0.03 (-0.06, 0.13) |
| Cerebrovascular disorders | 1356753 | 1389 | 83066 | 133 | 1.18 (0.96, 1.44) | 0.18 (0.10, 0.26) |
| Dysrhythmias | 1358757 | 1314 | 83294 | 135 | 1.40 (1.13, 1.74) | 0.39 (0.31, 0.47) |
| Ischemic heart diseases | 1352035 | 2240 | 82530 | 209 | 0.99 (0.84, 1.18) | -0.02 (-0.12, 0.07) |
| Other cardiac disorders | 1364629 | 626 | 83664 | 87 | 1.25 (0.97, 1.60) | 0.12 (0.07, 0.18) |
| Thrombotic disorders | 1365595 | 355 | 83811 | 36 | 1.21 (0.81, 1.83) | 0.05 (0.01, 0.09) |
| Inflammatory heart disease | 1367184 | 18 | 83962 | 3 | 2.32 (0.60, 8.98) | 0.02 (0.01, 0.03) |
| Stroke | 1358420 | 1115 | 83192 | 107 | 1.09 (0.87,1.36) | 0.07 (-0.00, 0.14) |
| TIA | 1364975 | 367 | 83781 | 34 | 1.38 (0.92, 2.09) | 0.10 (0.06, 0.14) |
| Atrial fibrillation | 1365241 | 407 | 83779 | 44 | 1.32 (0.91, 1.90) | 0.09 (0.05, 0.13) |
| Sinus tachycardia | 1365154 | 381 | 83802 | 25 | 0.79 (0.50, 1.25) | -0.06 (-0.10, -0.02) |
| Sinus bradycardia | 1366637 | 117 | 83914 | 18 | 1.64 (0.92, 2.93) | 0.06 (0.03, 0.08) |
| Other arrhythmia | 1363124 | 534 | 83673 | 56 | 1.71 (1.23, 2.38) | 0.28 (0.23, 0.34) |
| Myocardial infarction | 1361355 | 1152 | 83352 | 104 | 0.98 (0.76, 1.26) | -0.03 (-0.10, 0.04) |
| Acute coronary disease | 1356067 | 1623 | 82910 | 138 | 0.89 (0.73, 1.08) | -0.14 (-0.22, -0.06) |
| Ischemic cardiomyopathy | 1366918 | 96 | 83917 | 11 | 0.78 (0.40, 1.52) | -0.01 (-0.03, 0.00) |
| Angina | 1362750 | 429 | 83516 | 51 | 1.31 (0.92, 1.86) | 0.10 (0.05, 0.14) |
| Heart failure | 1365349 | 465 | 83744 | 61 | 1.11 (0.82, 1.49) | 0.04 (-0.00, 0.09) |
| Non ischemic cardiomyopathy | 1366300 | 153 | 83871 | 25 | 1.70 (1.06, 2.71) | 0.08 (0.05, 0.11) |
| Cardiac arrest | 1367316 | 74 | 83970 | 6 | 0.60 (0.25, 1.42) | -0.02 (-0.04, -0.01) |
| Cardiogenic shock | 1367288 | 36 | 83968 | 3 | 0.53 (0.16, 1.77) | -0.01 (-0.02, -0.00) |
| Pulmonary embolism | 1366910 | 136 | 83946 | 8 | 0.77 (0.35, 1.66) | -0.02 (-0.05, -0.00) |
| Deep venous thrombosis | 1366167 | 219 | 83859 | 24 | 1.06 (0.65, 1.72) | 0.01 (-0.02, 0.04) |
| Superficial venous thromboses | 1367143 | 40 | 83956 | 6 | 2.75 (0.98, 7.70) | 0.05 (0.04, 0.07) |
| Arterial thromboses | 1367233 | 16 | 83964 | 1 | 0.46 (0.27, 0.79) | -0.00 (-0.01, 0.01) |
| Pericarditis | 1367244 | 11 | 83965 | 3 | 3.84 (0.94, 15.6) | 0.02 (0.01, 0.03) |
| Myocarditis | 1367296 | 9 | 83974 | 0 | –^#^ | -0.01 (-0.01, -0.00) |

Hazards ratio (HR) and excess burdens of pre-specified cardiovascular complications in the COVID-19 and control groups. 95% confidence intervals are reported in parentheses.

*denotes 95% CIs which are bounded away from 0. ^#^not estimable due to 0 cases in either group.

HR > 1 denotes higher risk of a respective composite/individual new cardiovascular condition in the COVID-19 exposed group versus control group.

Excess burdens > 0 denotes excess burden in a respective composite/individual new cardiovascular condition in the COVID-19 exposed group versus control group

# Table 5: Subgroup analysis: hazards ratios and excess burdens per 1000 person years for age 66+

|  | No. of Individuals | | | |  |  |
| --- | --- | --- | --- | --- | --- | --- |
| Outcome | Controls | Controls with outcome | Cases | Cases with outcome | HR | Excess burden (weighted, per 1000 person years) |
| Any cardiovascular, cerebrovascular  and other thrombotic complications | 274035 | 5129 | 18252 | 464 | 1.14 (1.02, 1.28) | 2.90 (2.18, 3.63) |
| MACE | 300831 | 3305 | 20438 | 339 | 1.20 (1.04,1.37) | 2.25 (1.71, 2.78) |
| Cerebrovascular disorders | 298291 | 1887 | 20373 | 157 | 1.03 (0.85, 1.26) | 0.27 (-0.12, 0.67) |
| Dysrhythmias | 305805 | 1757 | 21114 | 176 | 1.24 (1.03, 1.49) | 1.44 (1.05, 1.83) |
| Ischemic heart diseases | 300189 | 2151 | 20553 | 183 | 1.02 (0.85, 1.21) | 0.18 (-0.23, 0.60) |
| Other cardiac disorders | 311558 | 1269 | 21468 | 149 | 1.35 (1.11, 1.65) | 1.52 (1.19, 1.86) |
| Thrombotic disorders | 314883 | 454 | 21851 | 48 | 1.16 (0.82, 1.64) | 0.25 (0.06, 0.44) |
| Inflammatory heart disease | 316701 | 5 | 22032 | 0 | –^#^ | -0.02 (-0.03, -0.00) |
| Stroke | 299907 | 1661 | 20496 | 136 | 1.01 (0.81,1.25) | 0.07 (-0.30, 0.43) |
| TIA | 313974 | 366 | 21803 | 29 | 1.01 (0.65, 1.57) | 0.02 (-0.14, 0.19) |
| Atrial fibrillation | 310397 | 1114 | 21480 | 98 | 1.08 (0.84, 1.39) | 0.37 (0.08, 0.67) |
| Sinus tachycardia | 315339 | 271 | 21914 | 33 | 1.35 (0.89, 2.05) | 0.31 (0.16, 0.46) |
| Sinus bradycardia | 315640 | 155 | 21945 | 21 | 1.50 (0.90, 2.50) | 0.25 (0.13, 0.37) |
| Other arrhythmia | 313344 | 472 | 21747 | 62 | 1.66 (1.23, 2.24) | 0.99 (0.78, 1.21) |
| Myocardial infarction | 310619 | 1302 | 21421 | 131 | 1.11 (0.90, 1.38) | 0.51 (0.18, 0.83) |
| Acute coronary disease | 305894 | 1259 | 21126 | 100 | 0.95 (0.75, 1.20) | -0.18 (-0.48, 0.13) |
| Ischemic cardiomyopathy | 316064 | 132 | 21949 | 10 | 1.32 (0.65, 2.67) | 0.14 (0.03, 0.25) |
| Angina | 310950 | 373 | 21522 | 36 | 1.07 (0.74, 1.57) | 0.10 (-0.07, 0.27) |
| Heart failure | 312145 | 1059 | 21526 | 127 | 1.34 (1.08, 1.66) | 1.23 (0.93, 1.54) |
| Non ischemic cardiomyopathy | 315894 | 144 | 21942 | 18 | 1.49 (0.84, 2.64) | 0.23 (0.12, 0.35) |
| Cardiac arrest | 316682 | 114 | 22030 | 13 | 1.58 (0.80, 3.14) | 0.21 (0.11, 0.32) |
| Cardiogenic shock | 316649 | 45 | 22032 | 5 | 0.93 (0.36, 2.43) | -0.01 (-0.07, 0.04) |
| Pulmonary embolism | 316229 | 121 | 21986 | 13 | 1.50 (0.77, 2.90) | 0.19 (0.09, 0.30) |
| Deep venous thrombosis | 315560 | 290 | 21908 | 32 | 1.05 (0.69, 1.60) | 0.06 (-0.09, 0.21) |
| Superficial venous thromboses | 316497 | 48 | 22014 | 4 | 1.02 (0.35, 2.97) | 0.00 (-0.06, 0.06) |
| Arterial thromboses | 316576 | 31 | 22026 | 2 | 0.59 (0.14, 2.47) | -0.04 (-0.08, 0.00) |
| Pericarditis | 316707 | 2 | 22033 | 0 | –^#^ | -0.01 (-0.01, 0.00) |
| Myocarditis | 316717 | 3 | 22034 | 0 | –^#^ | -0.01 (-0.02, 0.00) |

Hazards ratio (HR) and excess burdens of pre-specified cardiovascular complications in the COVID-19 and control groups. 95% confidence intervals are reported in parentheses.

*denotes 95% CIs which are bounded away from 0. ^#^not estimable due to 0 cases in either group.

HR > 1 denotes higher risk of a respective composite/individual new cardiovascular condition in the COVID-19 exposed group versus control group.

Excess burdens > 0 denotes excess burden in a respective composite/individual new cardiovascular condition in the COVID-19 exposed group versus control group

# Table 6: Subgroup analysis: hazards ratios and excess burdens per 1000 person years for unvaccinated

|  | No. of Individuals | | | |  |  |
| --- | --- | --- | --- | --- | --- | --- |
| Outcome | Controls | Controls with outcome | Cases | Cases with outcome | HR | Excess burden (weighted, per 1000 person years) |
| Any cardiovascular, cerebrovascular  and other thrombotic complications | 106137 | 797 | 8052 | 140 | 1.56(1.29,1.90) | 3.95 (3.14, 4.75) |
| MACE | 110065 | 507 | 8596 | 95 | 1.51(1.19,1.92) | 2.13 (1.51, 2.75) |
| Cerebrovascular disorders | 110361 | 288 | 8673 | 42 | 1.25(0.89,1.76) | 0.61 (0.16, 1.05) |
| Dysrhythmias | 111132 | 281 | 8841 | 66 | 2.04(1.53,2.72) | 2.58 (2.07, 3.08) |
| Ischemic heart diseases | 110475 | 330 | 8694 | 63 | 1.45(1.07,1.95) | 1.22 (0.72, 1.71) |
| Other cardiac disorders | 112040 | 204 | 8907 | 51 | 1.92(1.39,2.65) | 1.60 (1.19, 2.02) |
| Thrombotic disorders | 112716 | 84 | 9034 | 16 | 1.68(0.95,2.95) | 0.50 (0.24, 0.76) |
| Inflammatory heart disease | 113129 | 0 | 9104 | 0 | –^#^ | 0.00 (0.00, 0.00) |
| Stroke | 110624 | 257 | 8711 | 39 | 1.27(0.89,1.82) | 0.61 (0.18, 1.03) |
| TIA | 112702 | 51 | 9036 | 4 | 0.74(0.23,2.38) | -0.14 (-0.30, 0.02) |
| Atrial fibrillation | 112185 | 152 | 8962 | 32 | 1.65(1.11,2.46) | 0.84 (0.50, 1.18) |
| Sinus tachycardia | 112765 | 67 | 9060 | 17 | 2.59(1.39,4.81) | 0.92 (0.66, 1.18) |
| Sinus bradycardia | 112986 | 17 | 9082 | 8 | 4.58(1.49,14.07) | 0.42 (0.27, 0.58) |
| Other arrhythmia | 112384 | 86 | 9009 | 21 | 2.15(1.29,3.60) | 0.89 (0.61, 1.17) |
| Myocardial infarction | 111913 | 211 | 8907 | 45 | 1.42(0.99,2.03) | 0.68 (0.29, 1.06) |
| Acute coronary disease | 111474 | 172 | 8852 | 24 | 1.11(0.69,1.77) | 0.19 (-0.14, 0.52) |
| Ischemic cardiomyopathy | 113005 | 34 | 9064 | 6 | 1.34(0.52,3.46) | 0.09 (-0.06, 0.24) |
| Angina | 112296 | 45 | 8975 | 12 | 2.17(1.09,4.32) | 0.46 (0.26, 0.66) |
| Heart failure | 112169 | 164 | 8925 | 44 | 2.04(1.44,2.90) | 1.48 (1.10, 1.86) |
| Non ischemic cardiomyopathy | 112942 | 30 | 9077 | 8 | 1.67(0.72,3.83) | 0.17 (0.02, 0.32) |
| Cardiac arrest | 113133 | 31 | 9103 | 3 | 1.01(0.29,3.52) | 0.00 (-0.14, 0.14) |
| Cardiogenic shock | 113124 | 6 | 9105 | 1 | 0.61(0.22,1.68) | -0.02 (-0.07, 0.04) |
| Pulmonary embolism | 113030 | 25 | 9085 | 3 | 1.52(0.46,5.06) | 0.13 (-0.00, 0.27) |
| Deep venous thrombosis | 112866 | 50 | 9052 | 12 | 1.85(0.95,3.60) | 0.36 (0.15, 0.56) |
| Superficial venous thromboses | 113095 | 7 | 9101 | 1 | 1.47(0.21,10.35) | 0.03 (-0.04, 0.10) |
| Arterial thromboses | 113109 | 8 | 9102 | 1 | 1.30(0.45,3.75) | 0.05 (-0.03, 0.13) |
| Pericarditis | 113137 | 0 | 9105 | 0 | –^#^ | 0.00 (0.00, 0.00) |
| Myocarditis | 113136 | 0 | 9106 | 0 | –^#^ | 0.00 (0.00, 0.00) |

Hazards ratio (HR) and excess burdens of pre-specified cardiovascular complications in the COVID-19 and control groups. 95% confidence intervals are reported in parentheses.

*denotes 95% CIs which are bounded away from 0. ^#^not estimable due to 0 cases in either group.

HR > 1 denotes higher risk of a respective composite/individual new cardiovascular condition in the COVID-19 exposed group versus control group.

Excess burdens > 0 denotes excess burden in a respective composite/individual new cardiovascular condition in the COVID-19 exposed group versus control group

# Table 7: Subgroup analysis: hazards ratios and excess burdens per 1000 person years for fully vaccinated

|  | No. of Individuals |  |  |  |  |  |
| --- | --- | --- | --- | --- | --- | --- |
| Outcome | Controls | Controls with outcome | Cases | Cases with outcome | HR | Excess burden (weighted, per 1000 person years) |
| Any cardiovascular, cerebrovascular  and other thrombotic complications | 1222349 | 5952 | 80835 | 619 | 1.11(1.02,1.22) | 0.41 (0.24, 0.59) |
| MACE | 1251501 | 3293 | 83614 | 382 | 1.12(0.99,1.26) | 0.20 (0.07, 0.33) |
| Cerebrovascular disorders | 1251475 | 1912 | 83772 | 193 | 1.04(0.88,1.22) | 0.01 (-0.09, 0.10) |
| Dysrhythmias | 1256022 | 1869 | 84385 | 193 | 1.18(1.00,1.40) | 0.24 (0.14, 0.33) |
| Ischemic heart diseases | 1249648 | 2582 | 83474 | 262 | 1.00(0.87,1.16) | -0.03 (-0.14, 0.08) |
| Other cardiac disorders | 1263553 | 1070 | 84928 | 162 | 1.43(1.19,1.70) | 0.31 (0.24, 0.39) |
| Thrombotic disorders | 1265925 | 508 | 85270 | 60 | 1.30(0.95,1.79) | 0.11 (0.05, 0.16) |
| Inflammatory heart disease | 1268065 | 19 | 85487 | 3 | 2.18(0.57,8.36) | 0.02 (0.00, 0.03) |
| Stroke | 1253455 | 1615 | 83940 | 161 | 0.98(0.82,1.17) | -0.07 (-0.16, 0.01) |
| TIA | 1265163 | 428 | 85212 | 45 | 1.20(0.83,1.73) | 0.06 (0.02, 0.11) |
| Atrial fibrillation | 1263435 | 852 | 85006 | 88 | 1.14(0.90,1.44) | 0.05 (-0.01, 0.11) |
| Sinus tachycardia | 1265782 | 436 | 85295 | 35 | 0.82(0.56,1.19) | -0.07 (-0.11, -0.03) |
| Sinus bradycardia | 1267101 | 181 | 85397 | 26 | 1.40(0.87,2.25) | 0.05 (0.02, 0.08) |
| Other arrhythmia | 1263201 | 634 | 85071 | 71 | 1.40(1.05,1.86) | 0.20 (0.14, 0.26) |
| Myocardial infarction | 1260937 | 1458 | 84639 | 146 | 0.66(0.35,1.24) | -0.11 (-0.19, -0.03) |
| Acute coronary disease | 1255315 | 1703 | 84121 | 172 | 0.97(0.82,1.15) | -0.05 (-0.14, 0.03) |
| Ischemic cardiomyopathy | 1267590 | 124 | 85411 | 10 | 0.81(0.39,1.66) | -0.02 (-0.04, 0.01) |
| Angina | 1262246 | 497 | 84828 | 64 | 1.32(0.97,1.79) | 0.12 (0.07, 0.17) |
| Heart failure | 1264359 | 846 | 85028 | 130 | 1.45(1.19,1.77) | 0.25 (0.19, 0.32) |
| Non ischemic cardiomyopathy | 1267027 | 179 | 85355 | 28 | 1.48(0.98,2.23) | 0.07 (0.04, 0.10) |
| Cardiac arrest | 1268165 | 102 | 85497 | 13 | 0.75(0.10,5.95) | 0.00 (-0.02, 0.02) |
| Cardiogenic shock | 1268132 | 54 | 85491 | 7 | 1.01(0.45,2.27) | -0.00 (-0.02, 0.01) |
| Pulmonary embolism | 1267634 | 162 | 85451 | 13 | 0.83(0.45,1.52) | -0.03 (-0.05, -0.00) |
| Deep venous thrombosis | 1266716 | 323 | 85343 | 41 | 1.27(0.87,1.86) | 0.06 (0.02, 0.10) |
| Superficial venous thromboses | 1267932 | 56 | 85467 | 9 | 2.57(1.13,5.87) | 0.07 (0.05, 0.09) |
| Arterial thromboses | 1268056 | 27 | 85489 | 1 | 0.20(0.03,1.53) | -0.02 (-0.03, -0.01) |
| Pericarditis | 1268114 | 11 | 85489 | 3 | 0.00(0.00,0.00) | 0.02 (0.01, 0.03) |
| Myocarditis | 1268169 | 10 | 85498 | 0 | –^#^ | -0.01 (-0.01, -0.00) |

Hazards ratio (HR) and excess burdens of pre-specified cardiovascular complications in the COVID-19 and control groups. 95% confidence intervals are reported in parentheses.

*denotes 95% CIs which are bounded away from 0. ^#^not estimable due to 0 cases in either group.

HR > 1 denotes higher risk of a respective composite/individual new cardiovascular condition in the COVID-19 exposed group versus control group.

Excess burdens > 0 denotes excess burden in a respective composite/individual new cardiovascular condition in the COVID-19 exposed group versus control group

# Table 8: Subgroup analysis: hazards ratios and excess burdens per 1000 person years for fully vaccinated and boosted

|  | No. of Individuals | | | |  |  |
| --- | --- | --- | --- | --- | --- | --- |
| Outcome | Controls | Controls with outcome | Cases | Cases with outcome | HR | Excess burden (weighted, per 1000 person years) |
| Any cardiovascular, cerebrovascular  and other thrombotic complications | 278671 | 3221 | 10327 | 153 | 1.10(0.92,1.32) | 1.11 (0.55, 1.68) |
| MACE | 294598 | 1857 | 11005 | 83 | 1.05(0.81,1.35) | 0.27 (-0.13, 0.68) |
| Cerebrovascular disorders | 293208 | 1076 | 10994 | 55 | 1.20(0.89,1.62) | 0.75 (0.43, 1.07) |
| Dysrhythmias | 297408 | 921 | 11182 | 52 | 1.38(1.00,1.91) | 1.12 (0.82, 1.42) |
| Ischemic heart diseases | 292101 | 1479 | 10915 | 67 | 0.98(0.75,1.29) | -0.10 (-0.46, 0.26) |
| Other cardiac disorders | 300594 | 621 | 11297 | 23 | 0.93(0.59,1.46) | -0.16 (-0.38, 0.06) |
| Thrombotic disorders | 301837 | 217 | 11358 | 8 | 0.94(0.42,2.12) | -0.10 (-0.22, 0.03) |
| Inflammatory heart disease | 302691 | 4 | 11403 | 0 | –^#^ | -0.01 (-0.03, -0.00) |
| Stroke | 294248 | 904 | 11037 | 43 | 1.13(0.80,1.59) | 0.39 (0.10, 0.68) |
| TIA | 301084 | 254 | 11336 | 14 | 1.25(0.72,2.19) | 0.21 (0.06, 0.37) |
| Atrial fibrillation | 300018 | 517 | 11291 | 22 | 1.07(0.64,1.78) | 0.10 (-0.11, 0.31) |
| Sinus tachycardia | 301946 | 149 | 11361 | 6 | 0.95(0.40,2.26) | -0.03 (-0.14, 0.08) |
| Sinus bradycardia | 302190 | 74 | 11380 | 5 | 1.39(0.55,3.50) | 0.10 (0.01, 0.18) |
| Other arrhythmia | 300883 | 286 | 11340 | 26 | 2.20(1.40,3.46) | 1.13 (0.94, 1.32) |
| Myocardial infarction | 299124 | 785 | 11227 | 44 | 1.25(0.88,1.77) | 0.64 (0.37, 0.91) |
| Acute coronary disease | 295172 | 1007 | 11063 | 42 | 0.86(0.63,1.19) | -0.46 (-0.75, -0.18) |
| Ischemic cardiomyopathy | 302387 | 70 | 11391 | 5 | 1.65(0.63,4.32) | 0.13 (0.04, 0.22) |
| Angina | 299158 | 260 | 11235 | 11 | 0.82(0.44,1.51) | -0.15 (-0.29, -0.01) |
| Heart failure | 300966 | 514 | 11317 | 14 | 0.70(0.39,1.26) | -0.51 (-0.70, -0.32) |
| Non ischemic cardiomyopathy | 302225 | 88 | 11381 | 7 | 1.79(0.82,3.93) | 0.23 (0.13, 0.33) |
| Cardiac arrest | 302700 | 55 | 11400 | 3 | 1.58(0.45,5.63) | 0.10 (0.03, 0.18) |
| Cardiogenic shock | 302681 | 21 | 11404 | 0 | –^#^ | -0.07 (-0.10, -0.04) |
| Pulmonary embolism | 302475 | 70 | 11396 | 5 | 1.91(0.67,5.47) | 0.17 (0.08, 0.26) |
| Deep venous thrombosis | 302145 | 136 | 11372 | 3 | 0.47(0.14,1.60) | -0.25 (-0.34, -0.16) |
| Superficial venous thromboses | 302613 | 25 | 11402 | 0 | –^#^ | -0.08 (-0.11, -0.05) |
| Arterial thromboses | 302644 | 12 | 11399 | 1 | 0.93(0.51,1.69) | 0.03 (-0.01, 0.07) |
| Pericarditis | 302700 | 2 | 11404 | 0 | –^#^ | -0.01 (-0.02, 0.00) |
| Myocarditis | 302708 | 2 | 11404 | 0 | –^#^ | -0.01 (-0.02, 0.00) |

Hazards ratio (HR) and excess burdens of pre-specified cardiovascular complications in the COVID-19 and control groups. 95% confidence intervals are reported in parentheses.

*denotes 95% CIs which are bounded away from 0. ^#^not estimable due to 0 cases in either group.

HR > 1 denotes higher risk of a respective composite/individual new cardiovascular condition in the COVID-19 exposed group versus control group.

Excess burdens > 0 denotes excess burden in a respective composite/individual new cardiovascular condition in the COVID-19 exposed group versus control group

# Table 9: Subgroup analysis: hazards ratios and excess burdens per 1000 person years for Chinese

| Outcome | Controls | Controls with outcome | Cases | Cases with outcome | HR | Excess burden (weighted, per 1000 person years) |
| --- | --- | --- | --- | --- | --- | --- |
| Any cardiovascular, cerebrovascular  and other thrombotic complications | 1216582 | 7432 | 67636 | 616 | 1.13 (1.03, 1.25) | 0.55 (0.36, 0.75) |
| MACE | 1254840 | 4082 | 70473 | 387 | 1.17 (1.03, 1.32) | 0.36 (0.21, 0.50) |
| Cerebrovascular disorders | 1251434 | 2548 | 70372 | 204 | 1.08 (0.91, 1.28) | 0.07 (-0.04, 0.18) |
| Dysrhythmias | 1259420 | 2403 | 71125 | 220 | 1.29 (1.09, 1.52) | 0.46 (0.35, 0.57) |
| Ischemic heart diseases | 1251737 | 3097 | 70410 | 243 | 1.03 (0.88, 1.20) | -0.00 (-0.13, 0.12) |
| Other cardiac disorders | 1269083 | 1337 | 71713 | 165 | 1.43 (1.19, 1.71) | 0.38 (0.30, 0.47) |
| Thrombotic disorders | 1272058 | 588 | 72020 | 56 | 1.14 (0.83, 1.57) | 0.05 (0.00, 0.11) |
| Inflammatory heart disease | 1274377 | 17 | 72245 | 2 | 1.92 (1.92, 1.92) | 0.01 (0.00, 0.02) |
| Stroke | 1253960 | 2161 | 70533 | 175 | 1.03(0.85,1.23) | -0.05 (-0.15, 0.05) |
| TIA | 1270534 | 566 | 71965 | 41 | 1.15 (0.79, 1.67) | 0.05 (0.00, 0.11) |
| Atrial fibrillation | 1267749 | 1228 | 71696 | 104 | 1.09 (0.86, 1.37) | 0.01 (-0.07, 0.08) |
| Sinus tachycardia | 1271899 | 476 | 72061 | 39 | 0.98 (0.67, 1.42) | -0.01 (-0.06, 0.03) |
| Sinus bradycardia | 1273209 | 196 | 72154 | 26 | 1.67 (1.05, 2.65) | 0.09 (0.06, 0.13) |
| Other arrhythmia | 1268575 | 802 | 71841 | 88 | 1.74 (1.33, 2.26) | 0.45 (0.38, 0.52) |
| Myocardial infarction | 1266635 | 1628 | 71544 | 151 | 1.16 (0.94, 1.42) | 0.14 (0.05, 0.23) |
| Acute coronary disease | 1258844 | 2005 | 71038 | 145 | 0.94 (0.78, 1.14) | -0.12 (-0.21, -0.02) |
| Ischemic cardiomyopathy | 1273826 | 146 | 72155 | 15 | 1.37 (0.77, 2.45) | 0.04 (0.01, 0.07) |
| Angina | 1266938 | 547 | 71633 | 42 | 1.07 (0.75, 1.54) | 0.02 (-0.03, 0.07) |
| Heart failure | 1270042 | 1048 | 71789 | 128 | 1.37 (1.11, 1.68) | 0.24 (0.16, 0.31) |
| Non ischemic cardiomyopathy | 1273162 | 220 | 72137 | 30 | 1.76 (1.16, 2.67) | 0.13 (0.09, 0.17) |
| Cardiac arrest | 1274441 | 144 | 72246 | 15 | 1.26 (0.68, 2.34) | 0.02 (-0.00, 0.05) |
| Cardiogenic shock | 1274407 | 54 | 72245 | 6 | 0.91 (0.38, 2.17) | -0.01 (-0.02, 0.01) |
| Pulmonary embolism | 1273877 | 184 | 72192 | 13 | 0.89 (0.48, 1.64) | -0.02 (-0.05, 0.01) |
| Deep venous thrombosis | 1272914 | 367 | 72095 | 38 | 1.10 (0.76, 1.62) | 0.02 (-0.02, 0.06) |
| Superficial venous thromboses | 1274219 | 60 | 72228 | 6 | 1.94 (0.73, 5.17) | 0.04 (0.02, 0.06) |
| Arterial thromboses | 1274326 | 37 | 72239 | 0 | –^#^ | -0.03 (-0.04, -0.02) |
| Pericarditis | 1274418 | 8 | 72247 | 2 | 4.20 (0.78, 22.3) | 0.02 (0.01, 0.03) |
| Myocarditis | 1274465 | 10 | 72250 | 0 | –^#^ | -0.01 (-0.01, -0.00) |

Hazards ratio (HR) and excess burdens of pre-specified cardiovascular complications in the COVID-19 and control groups. 95% confidence intervals are reported in parentheses.

*denotes 95% CIs which are bounded away from 0. ^#^not estimable due to 0 cases in either group.

HR > 1 denotes higher risk of a respective composite/individual new cardiovascular condition in the COVID-19 exposed group versus control group.

Excess burdens > 0 denotes excess burden in a respective composite/individual new cardiovascular condition in the COVID-19 exposed group versus control group

# Table 10: Subgroup analysis: hazards ratios and excess burdens per 1000 person years for Malay

| Outcome | Controls | Controls with outcome | Cases | Cases with outcome | HR | Excess burden (weighted, per 1000 person years) |
| --- | --- | --- | --- | --- | --- | --- |
| Any cardiovascular, cerebrovascular  and other thrombotic complications | 187505 | 1339 | 17839 | 180 | 1.27 (1.07, 1.50) | 1.55 (1.00, 2.09) |
| MACE | 192641 | 882 | 18423 | 97 | 1.01 (0.80, 1.27) | -0.23 (-0.64, 0.17) |
| Cerebrovascular disorders | 193885 | 430 | 18594 | 54 | 1.24 (0.91, 1.69) | 0.40 (0.10, 0.70) |
| Dysrhythmias | 194775 | 365 | 18708 | 61 | 1.58 (1.18, 2.11) | 1.00 (0.70, 1.29) |
| Ischemic heart diseases | 192912 | 644 | 18454 | 77 | 1.04 (0.81, 1.33) | 0.05 (-0.30, 0.40) |
| Other cardiac disorders | 195750 | 338 | 18782 | 42 | 1.02 (0.72, 1.43) | -0.09 (-0.33, 0.16) |
| Thrombotic disorders | 196487 | 129 | 18905 | 18 | 1.22 (0.70, 2.14) | 0.12 (-0.04, 0.28) |
| Inflammatory heart disease | 197087 | 1 | 18966 | 1 | 9.16 (9.16, 9.16) | 0.03 (0.01, 0.06) |
| Stroke | 194200 | 374 | 18636 | 42 | 1.11(0.78,1.58) | 0.10 (-0.17, 0.37) |
| TIA | 196614 | 79 | 18900 | 15 | 1.98(1.10, 3.56) | 0.37 (0.22, 0.51) |
| Atrial fibrillation | 196064 | 195 | 18843 | 26 | 1.32 (0.84, 2.07) | 0.21 (0.01, 0.41) |
| Sinus tachycardia | 196656 | 84 | 18920 | 11 | 1.08 (0.57, 2.08) | 0.04 (-0.09, 0.17) |
| Sinus bradycardia | 196846 | 48 | 18938 | 9 | 1.78 (0.83, 3.83) | 0.19 (0.08, 0.30) |
| Other arrhythmia | 196351 | 88 | 18888 | 20 | 2.13 (1.27, 3.56) | 0.48 (0.33, 0.64) |
| Myocardial infarction | 194937 | 421 | 18698 | 38 | 0.78 (0.55, 1.12) | -0.53 (-0.79, -0.26) |
| Acute coronary disease | 194157 | 430 | 18592 | 51 | 1.07 (0.79, 1.46) | 0.14 (-0.15, 0.43) |
| Ischemic cardiomyopathy | 196890 | 59 | 18946 | 4 | 0.52 (0.16, 1.61) | -0.14 (-0.24, -0.05) |
| Angina | 195904 | 117 | 18816 | 22 | 1.68 (1.04, 2.73) | 0.39 (0.22, 0.56) |
| Heart failure | 195936 | 289 | 18817 | 34 | 0.94 (0.64, 1.37) | -0.18 (-0.41, 0.04) |
| Non ischemic cardiomyopathy | 196820 | 46 | 18927 | 9 | 1.46 (0.69, 3.05) | 0.10 (-0.00, 0.20) |
| Cardiac arrest | 197112 | 27 | 18968 | 4 | 1.25 (0.40, 3.90) | 0.03 (-0.04, 0.10) |
| Cardiogenic shock | 197096 | 15 | 18968 | 2 | 0.81 (0.18,3.58) | -0.01 (-0.06, 0.04) |
| Pulmonary embolism | 196948 | 39 | 18958 | 4 | 1.38 (0.43, 4.38) | 0.07 (-0.02, 0.16) |
| Deep venous thrombosis | 196704 | 84 | 18922 | 14 | 1.27 (0.69, 2.32) | 0.11 (-0.02, 0.24) |
| Superficial venous thromboses | 197045 | 17 | 18961 | 3 | 1.65(0.47, 5.71) | 0.05 (-0.01, 0.11) |
| Arterial thromboses | 197066 | 5 | 18968 | 1 | 1.04(0.11, 9.57) | 0.00 (-0.03, 0.03) |
| Pericarditis | 197104 | 1 | 18966 | 1 | 9.16(9.16, 9.16) | 0.03 (0.01, 0.06) |
| Myocarditis | 197102 | 0 | 18971 | 0 | –^#^ | 0.00 (0.00, 0.00) |

Hazards ratio (HR) and excess burdens of pre-specified cardiovascular complications in the COVID-19 and control groups. 95% confidence intervals are reported in parentheses.

*denotes 95% CIs which are bounded away from 0. ^#^not estimable due to 0 cases in either group.

HR > 1 denotes higher risk of a respective composite/individual new cardiovascular condition in the COVID-19 exposed group versus control group.

Excess burdens > 0 denotes excess burden in a respective composite/individual new cardiovascular condition in the COVID-19 exposed group versus control group

# Table 11: Subgroup analysis: hazards ratios and excess burdens per 1000 person years for Indian

| Outcome | Controls | Controls with outcome | Cases | Cases with outcome | HR | Excess burden (weighted, per 1000 person years) |
| --- | --- | --- | --- | --- | --- | --- |
| Any cardiovascular, cerebrovascular  and other thrombotic complications | 148562 | 1002 | 11036 | 101 | 1.09 (0.86, 1.37) | 0.30 (-0.28, 0.88) |
| MACE | 153097 | 614 | 11537 | 71 | 1.05 (0.79, 1.37) | -0.00 (-0.44, 0.44) |
| Cerebrovascular disorders | 154132 | 255 | 11684 | 29 | 1.28 (0.84, 1.95) | 0.41 (0.11, 0.71) |
| Dysrhythmias | 154832 | 236 | 11792 | 21 | 0.92 (0.54, 1.55) | -0.15 (-0.41, 0.11) |
| Ischemic heart diseases | 152162 | 572 | 11445 | 68 | 1.05 (0.80, 1.38) | 0.10 (-0.33, 0.53) |
| Other cardiac disorders | 155462 | 191 | 11828 | 27 | 1.15 (0.72, 1.82) | 0.13 (-0.12, 0.38) |
| Thrombotic disorders | 156022 | 75 | 11921 | 10 | 1.94 (0.90, 4.18) | 0.45 (0.27, 0.63) |
| Inflammatory heart disease | 156408 | 3 | 11959 | 0 | –^#^ | -0.02 (-0.04, 0.00) |
| Stroke | 154487 | 209 | 11722 | 23 | 1.19(0.75,1.88) | 0.20 (-0.06, 0.46) |
| TIA | 155895 | 70 | 11905 | 7 | 1.23 (0.50, 3.04) | 0.10 (-0.05, 0.26) |
| Atrial fibrillation | 156007 | 64 | 11908 | 6 | 0.72 (0.29, 1.76) | -0.14 (-0.27, -0.01) |
| Sinus tachycardia | 156004 | 76 | 11920 | 6 | 1.05 (0.38, 2.85) | 0.02 (-0.13, 0.17) |
| Sinus bradycardia | 156246 | 21 | 11949 | 4 | 1.31 (0.43, 3.94) | 0.04 (-0.04, 0.13) |
| Other arrhythmia | 155723 | 101 | 11888 | 8 | 0.85 (0.38, 1.88) | -0.11 (-0.27, 0.06) |
| Myocardial infarction | 154554 | 369 | 11727 | 45 | 1.05 (0.74, 1.48) | 0.05 (-0.29, 0.39) |
| Acute coronary disease | 153405 | 391 | 11622 | 39 | 0.83 (0.59, 1.19) | -0.47 (-0.80, -0.14) |
| Ischemic cardiomyopathy | 156267 | 21 | 11943 | 2 | 0.89 (0.16, 4.93) | -0.02 (-0.10, 0.05) |
| Angina | 155011 | 120 | 11778 | 22 | 1.54 (0.95, 2.50) | 0.41 (0.19, 0.62) |
| Heart failure | 155602 | 162 | 11852 | 24 | 1.24 (0.75, 2.03) | 0.20 (-0.03, 0.43) |
| Non ischemic cardiomyopathy | 156232 | 23 | 11929 | 4 | 1.17 (0.39, 3.50) | 0.03 (-0.06, 0.11) |
| Cardiac arrest | 156426 | 16 | 11963 | 0 | –^#^ | -0.11 (-0.16, -0.06) |
| Cardiogenic shock | 156416 | 12 | 11963 | 0 | –^#^ | -0.08 (-0.13, -0.04) |
| Pulmonary embolism | 156329 | 27 | 11960 | 4 | 2.58 (0.79, 8.40) | 0.25 (0.13, 0.36) |
| Deep venous thrombosis | 156158 | 46 | 11931 | 4 | 1.06 (0.32, 3.50) | 0.04 (-0.08, 0.16) |
| Superficial venous thromboses | 156371 | 11 | 11960 | 1 | 1.14 (1.14, 1.14) | 0.00 (-0.05, 0.06) |
| Arterial thromboses | 156402 | 4 | 11961 | 2 | 8.11 (1.30, 50.34) | 0.20 (0.12, 0.27) |
| Pericarditis | 156415 | 3 | 11961 | 0 | –^#^ | -0.02 (-0.04, 0.00) |
| Myocarditis | 156427 | 0 | 11963 | 0 | –^#^ | 0.00 (0.00, 0.00) |

Hazards ratio (HR) and excess burdens of pre-specified cardiovascular complications in the COVID-19 and control groups. 95% confidence intervals are reported in parentheses.

*denotes 95% CIs which are bounded away from 0. ^#^not estimable due to 0 cases in either group.

HR > 1 denotes higher risk of a respective composite/individual new cardiovascular condition in the COVID-19 exposed group versus control group.

Excess burdens > 0 denotes excess burden in a respective composite/individual new cardiovascular condition in the COVID-19 exposed group versus control group

# Table 12: Subgroup analysis: hazards ratios and excess burdens per 1000 person years for Other Ethnicities

| Outcome | Controls | Controls with outcome | Cases | Cases with outcome | HR | Excess burden (weighted, per 1000 person years) |
| --- | --- | --- | --- | --- | --- | --- |
| Any cardiovascular, cerebrovascular  and other thrombotic complications | 54508 | 197 | 2703 | 15 | 1.30 (0.73, 2.30) | 0.94 (0.20, 1.68) |
| MACE | 55586 | 79 | 2782 | 5 | 0.98 (0.34, 2.82) | -0.09 (-0.52, 0.34) |
| Cerebrovascular disorders | 55593 | 43 | 2789 | 3 | 1.00 (0.28, 3.48) | -0.03 (-0.35, 0.29) |
| Dysrhythmias | 55535 | 67 | 2783 | 9 | 2.29 (1.10, 4.76) | 1.51 (1.00, 2.02) |
| Ischemic heart diseases | 55413 | 78 | 2774 | 4 | 1.02 (0.35, 2.98) | 0.14 (-0.30, 0.58) |
| Other cardiac disorders | 55892 | 29 | 2809 | 2 | 0.76 (0.17, 3.30) | -0.16 (-0.40, 0.08) |
| Thrombotic disorders | 55911 | 17 | 2816 | 0 | –^#^ | -0.31 (-0.45, -0.17) |
| Inflammatory heart disease | 56013 | 2 | 2824 | 0 | –^#^ | -0.03 (-0.08, 0.01) |
| Stroke | 55680 | 32 | 2797 | 3 | 1.35(0.37,4.81) | 0.17 (-0.13, 0.46) |
| TIA | 55906 | 18 | 2814 | 0 |  | -0.32 (-0.47, -0.18) |
| Atrial fibrillation | 55818 | 34 | 2812 | 6 | 2.87 (1.17, 7.03) | 1.25 (0.84, 1.65) |
| Sinus tachycardia | 55934 | 16 | 2815 | 2 | 1.25 (0.30, 5.12) | 0.13 (-0.08, 0.35) |
| Sinus bradycardia | 55976 | 7 | 2818 | 0 |  | -0.13 (-0.22, -0.04) |
| Other arrhythmia | 55819 | 15 | 2803 | 2 | 2.34 (0.39, 14.16) | 0.26 (0.03, 0.49) |
| Myocardial infarction | 55848 | 36 | 2804 | 1 | 0.85 (0.11, 6.31) | -0.12 (-0.40, 0.16) |
| Acute coronary disease | 55555 | 56 | 2784 | 3 | 0.82 (0.29, 2.32) | -0.00 (-0.37, 0.36) |
| Ischemic cardiomyopathy | 55999 | 2 | 2822 | 0 | –^#^ | -0.04 (-0.09, 0.01) |
| Angina | 55847 | 18 | 2811 | 1 | 3.10 (0.23, 41.37) | 0.35 (0.10, 0.61) |
| Heart failure | 55914 | 25 | 2812 | 2 | 0.86 (0.19, 3.76) | -0.10 (-0.33, 0.13) |
| Non ischemic cardiomyopathy | 55980 | 8 | 2820 | 0 | –^#^ | -0.15 (-0.24, -0.05) |
| Cardiac arrest | 56019 | 1 | 2823 | 0 | –^#^ | -0.02 (-0.05, 0.02) |
| Cardiogenic shock | 56018 | 0 | 2824 | 0 | –^#^ | 0.00 (0.00, 0.00) |
| Pulmonary embolism | 55985 | 7 | 2822 | 0 | –^#^ | -0.13 (-0.22, -0.04) |
| Deep venous thrombosis | 55951 | 12 | 2819 | 0 | –^#^ | -0.22 (-0.34, -0.10) |
| Superficial venous thromboses | 56005 | 0 | 2821 | 0 | –^#^ | 0.00 (0.00, 0.00) |
| Arterial thromboses | 56015 | 1 | 2822 | 0 | –^#^ | -0.02 (-0.05, 0.02) |
| Pericarditis | 56014 | 1 | 2824 | 0 | –^#^ | -0.02 (-0.05, 0.02) |
| Myocarditis | 56019 | 2 | 2824 | 0 | –^#^ | -0.03 (-0.08, 0.01) |

Hazards ratio (HR) and excess burdens of pre-specified cardiovascular complications in the COVID-19 and control groups. 95% confidence intervals are reported in parentheses.

*denotes 95% CIs which are bounded away from 0. ^#^not estimable due to 0 cases in either group.

HR > 1 denotes higher risk of a respective composite/individual new cardiovascular condition in the COVID-19 exposed group versus control group.

Excess burdens > 0 denotes excess burden in a respective composite/individual new cardiovascular condition in the COVID-19 exposed group versus control group

# Table 13: Main analysis with inverse probability weights without covariates in regression specification: hazards ratios and excess burdens per 1000 person years

|  | No. of Individuals | | | |  |  |
| --- | --- | --- | --- | --- | --- | --- |
| Outcome | Controls | Controls with outcome | Cases | Cases with outcome | HR (no cov) | Excess burden (weighted, per 1000 persons) |
| Any cardiovascular, cerebrovascular  and other thrombotic complications | 1607157 | 9970 | 99214 | 912 | 1.11(1.03-1.20) | 0.70 (0.53, 0.88) |
| MACE | 1656164 | 5657 | 103215 | 560 | 1.08(0.97-1.19) | 0.28 (0.15, 0.40) |
| Cerebrovascular disorders | 1655044 | 3276 | 103439 | 290 | 1.07(0.93-1.23) | 0.15 (0.05, 0.24) |
| Dysrhythmias | 1664562 | 3071 | 104408 | 311 | 1.27(1.11-1.46) | 0.51 (0.42, 0.61) |
| Ischemic heart diseases | 1652224 | 4391 | 103083 | 392 | 1.01(0.89-1.14) | 0.02 (-0.08, 0.13) |
| Other cardiac disorders | 1676187 | 1895 | 105132 | 236 | 1.25(1.08-1.46) | 0.30 (0.22, 0.37) |
| Thrombotic disorders | 1680478 | 809 | 105662 | 84 | 1.17(0.90-1.52) | 0.08 (0.04, 0.13) |
| Inflammatory heart disease | 1683885 | 23 | 105994 | 3 | 1.67(0.47-5.99) | 0.01 (0.00, 0.02) |
| Stroke | 1658327 | 2776 | 103688 | 243 | 1.01(0.87-1.17) | 0.02 (-0.07, 0.10) |
| TIA | 1678949 | 733 | 105584 | 63 | 1.18(0.87-1.60) | 0.08 (0.04, 0.13) |
| Atrial fibrillation | 1675638 | 1521 | 105259 | 142 | 1.07(0.88-1.30) | 0.06 (-0.00, 0.13) |
| Sinus tachycardia | 1680493 | 652 | 105716 | 58 | 1.02(0.75-1.38) | 0.01 (-0.03, 0.05) |
| Sinus bradycardia | 1682277 | 272 | 105859 | 39 | 1.59(1.09-2.34) | 0.10 (0.07, 0.13) |
| Other arrhythmia | 1676468 | 1006 | 105420 | 118 | 1.65(1.31-2.08) | 0.39 (0.33, 0.45) |
| Myocardial infarction | 1671974 | 2454 | 104773 | 235 | 1.04(0.88-1.22) | 0.06 (-0.03, 0.14) |
| Acute coronary disease | 1661961 | 2882 | 104036 | 238 | 0.94(0.81-1.09) | -0.11 (-0.19, -0.02) |
| Ischemic cardiomyopathy | 1682982 | 228 | 105866 | 21 | 1.08(0.65-1.78) | 0.01 (-0.01, 0.04) |
| Angina | 1673700 | 802 | 105038 | 87 | 1.22(0.94-1.57) | 0.11 (0.06, 0.15) |
| Heart failure | 1677494 | 1524 | 105270 | 188 | 1.19(1.00-1.41) | 0.18 (0.11, 0.25) |
| Non ischemic cardiomyopathy | 1682194 | 297 | 105813 | 43 | 1.60(1.12-2.29) | 0.11 (0.08, 0.14) |
| Cardiac arrest | 1683998 | 188 | 106000 | 19 | 1.10(0.65-1.85) | 0.01 (-0.01, 0.03) |
| Cardiogenic shock | 1683937 | 81 | 106000 | 8 | 0.73(0.35-1.52) | -0.01 (-0.03, -0.00) |
| Pulmonary embolism | 1683139 | 257 | 105932 | 21 | 1.07(0.65-1.75) | 0.01 (-0.02, 0.04) |
| Deep venous thrombosis | 1681727 | 509 | 105767 | 56 | 1.09(0.79-1.49) | 0.03 (-0.01, 0.06) |
| Superficial venous thromboses | 1683640 | 88 | 105970 | 10 | 1.75(0.79-3.86) | 0.04 (0.02, 0.06) |
| Arterial thromboses | 1683809 | 47 | 105990 | 3 | 0.74(0.21-2.59) | -0.01 (-0.02, 0.00) |
| Pericarditis | 1683951 | 13 | 105998 | 3 | 2.96(0.79-11.11) | 0.02 (0.01, 0.02) |
| Myocarditis | 1684013 | 12 | 106008 | 0 | –^#^ | -0.01 (-0.01, -0.00) |

Hazards ratio (HR) and excess burdens of pre-specified cardiovascular complications in the COVID-19 and control groups. 95% confidence intervals are reported in parentheses.

*denotes 95% CIs which are bounded away from 0. ^#^not estimable due to 0 cases in either group.

HR > 1 denotes higher risk of a respective composite/individual new cardiovascular condition in the COVID-19 exposed group versus control group.

Excess burdens > 0 denotes excess burden in a respective composite/individual new cardiovascular condition in the COVID-19 exposed group versus control group

# Table 14: Subgroup analysis with inverse probability weights without covariates in regression specification: hazards ratios and excess burdens per 1000 person years for unvaccinated

|  | No. of Individuals | | | |  |  |
| --- | --- | --- | --- | --- | --- | --- |
| Outcome | Controls | Controls with outcome | Cases | Cases with outcome | HR (no cov) | Excess burden (weighted, per 1000 person years) |
| Any cardiovascular, cerebrovascular  and other thrombotic complications | 106137 | 797 | 8052 | 140 | 1.51(1.24-1.83) | 3.95 (3.14, 4.75) |
| MACE | 110065 | 507 | 8596 | 95 | 1.44(1.14-1.82) | 2.13 (1.51, 2.75) |
| Cerebrovascular disorders | 110361 | 288 | 8673 | 42 | 1.22(0.86-1.73) | 0.61 (0.16, 1.05) |
| Dysrhythmias | 111132 | 281 | 8841 | 66 | 1.98(1.48-2.64) | 2.58 (2.07, 3.08) |
| Ischemic heart diseases | 110475 | 330 | 8694 | 63 | 1.39(1.03-1.87) | 1.22 (0.72, 1.71) |
| Other cardiac disorders | 112040 | 204 | 8907 | 51 | 1.83(1.32-2.54) | 1.60 (1.19, 2.02) |
| Thrombotic disorders | 112716 | 84 | 9034 | 16 | 1.63(0.93-2.87) | 0.50 (0.24, 0.76) |
| Inflammatory heart disease | 113129 | 0 | 9104 | 0 | –^#^ | 0.00 (0.00, 0.00) |
| Stroke | 110624 | 257 | 8711 | 39 | 1.25(0.87-1.79) | 0.61 (0.18, 1.03) |
| TIA | 112702 | 51 | 9036 | 4 | 0.70(0.22-2.17) | -0.14 (-0.30, 0.02) |
| Atrial fibrillation | 112185 | 152 | 8962 | 32 | 1.59(1.06-2.38) | 0.84 (0.50, 1.18) |
| Sinus tachycardia | 112765 | 67 | 9060 | 17 | 2.51(1.36-4.65) | 0.92 (0.66, 1.18) |
| Sinus bradycardia | 112986 | 17 | 9082 | 8 | 3.66(1.52-8.80) | 0.42 (0.27, 0.58) |
| Other arrhythmia | 112384 | 86 | 9009 | 21 | 2.13(1.28-3.53) | 0.89 (0.61, 1.17) |
| Myocardial infarction | 111913 | 211 | 8907 | 45 | 1.34(0.94-1.91) | 0.68 (0.29, 1.06) |
| Acute coronary disease | 111474 | 172 | 8852 | 24 | 1.12(0.70-1.78) | 0.19 (-0.14, 0.52) |
| Ischemic cardiomyopathy | 113005 | 34 | 9064 | 6 | 1.27(0.50-3.27) | 0.09 (-0.06, 0.24) |
| Angina | 112296 | 45 | 8975 | 12 | 2.08(1.07-4.05) | 0.46 (0.26, 0.66) |
| Heart failure | 112169 | 164 | 8925 | 44 | 1.95(1.37-2.79) | 1.48 (1.10, 1.86) |
| Non ischemic cardiomyopathy | 112942 | 30 | 9077 | 8 | 1.60(0.71-3.61) | 0.17 (0.02, 0.32) |
| Cardiac arrest | 113133 | 31 | 9103 | 3 | 1.00(0.30-3.32) | 0.00 (-0.14, 0.14) |
| Cardiogenic shock | 113124 | 6 | 9105 | 1 | 0.73(0.09-6.07) | -0.02 (-0.07, 0.04) |
| Pulmonary embolism | 113030 | 25 | 9085 | 3 | 1.58(0.46-5.40) | 0.13 (-0.00, 0.27) |
| Deep venous thrombosis | 112866 | 50 | 9052 | 12 | 1.75(0.91-3.37) | 0.36 (0.15, 0.56) |
| Superficial venous thromboses | 113095 | 7 | 9101 | 1 | 1.45(0.18-11.84) | 0.03 (-0.04, 0.10) |
| Arterial thromboses | 113109 | 8 | 9102 | 1 | 1.65(0.21-13.23) | 0.05 (-0.03, 0.13) |
| Pericarditis | 113137 | 0 | 9105 | 0 | –^#^ | 0.00 (0.00, 0.00) |
| Myocarditis | 113136 | 0 | 9106 | 0 | –^#^ | 0.00 (0.00, 0.00) |

Hazards ratio (HR) and excess burdens of pre-specified cardiovascular complications in the COVID-19 and control groups. 95% confidence intervals are reported in parentheses.

*denotes 95% CIs which are bounded away from 0. ^#^not estimable due to 0 cases in either group.

HR > 1 denotes higher risk of a respective composite/individual new cardiovascular condition in the COVID-19 exposed group versus control group.

Excess burdens > 0 denotes excess burden in a respective composite/individual new cardiovascular condition in the COVID-19 exposed group versus control group

# Table 15: Subgroup analysis with inverse probability weights without covariates in regression specification: hazards ratios and excess burdens per 1000 person years for fully vaccinated

|  | No. of Individuals | | | |  |  |
| --- | --- | --- | --- | --- | --- | --- |
| Outcome | Controls | Controls with outcome | Cases | Cases with outcome | HR (no cov) | Excess burden (weighted, per 1000 person years) |
| Any cardiovascular, cerebrovascular  and other thrombotic complications | 1222349 | 5952 | 80835 | 619 | 1.08(0.99-1.19) | 0.41 (0.24, 0.59) |
| MACE | 1251501 | 3293 | 83614 | 382 | 1.07(0.95-1.20) | 0.20 (0.07, 0.33) |
| Cerebrovascular disorders | 1251475 | 1912 | 83772 | 193 | 1.00(0.85-1.19) | 0.01 (-0.09, 0.10) |
| Dysrhythmias | 1256022 | 1869 | 84385 | 193 | 1.16(0.97-1.37) | 0.24 (0.14, 0.33) |
| Ischemic heart diseases | 1249648 | 2582 | 83474 | 262 | 0.99(0.86-1.14) | -0.03 (-0.14, 0.08) |
| Other cardiac disorders | 1263553 | 1070 | 84928 | 162 | 1.36(1.13-1.62) | 0.31 (0.24, 0.39) |
| Thrombotic disorders | 1265925 | 508 | 85270 | 60 | 1.26(0.92-1.73) | 0.11 (0.05, 0.16) |
| Inflammatory heart disease | 1268065 | 19 | 85487 | 3 | 2.06(0.57-7.49) | 0.02 (0.00, 0.03) |
| Stroke | 1253455 | 1615 | 83940 | 161 | 0.94(0.79-1.13) | -0.07 (-0.16, 0.01) |
| TIA | 1265163 | 428 | 85212 | 45 | 1.19(0.82-1.72) | 0.06 (0.02, 0.11) |
| Atrial fibrillation | 1263435 | 852 | 85006 | 88 | 1.07(0.84-1.37) | 0.05 (-0.01, 0.11) |
| Sinus tachycardia | 1265782 | 436 | 85295 | 35 | 0.81(0.56-1.17) | -0.07 (-0.11, -0.03) |
| Sinus bradycardia | 1267101 | 181 | 85397 | 26 | 1.35(0.84-2.16) | 0.05 (0.02, 0.08) |
| Other arrhythmia | 1263201 | 634 | 85071 | 71 | 1.39(1.04-1.86) | 0.20 (0.14, 0.26) |
| Myocardial infarction | 1260937 | 1458 | 84639 | 146 | 0.91(0.75-1.10) | -0.11 (-0.19, -0.03) |
| Acute coronary disease | 1255315 | 1703 | 84121 | 172 | 0.96(0.81-1.14) | -0.05 (-0.14, 0.03) |
| Ischemic cardiomyopathy | 1267590 | 124 | 85411 | 10 | 0.83(0.40-1.74) | -0.02 (-0.04, 0.01) |
| Angina | 1262246 | 497 | 84828 | 64 | 1.30(0.96-1.76) | 0.12 (0.07, 0.17) |
| Heart failure | 1264359 | 846 | 85028 | 130 | 1.36(1.11-1.67) | 0.25 (0.19, 0.32) |
| Non ischemic cardiomyopathy | 1267027 | 179 | 85355 | 28 | 1.47(0.97-2.23) | 0.07 (0.04, 0.10) |
| Cardiac arrest | 1268165 | 102 | 85497 | 13 | 1.02(0.56-1.84) | 0.00 (-0.02, 0.02) |
| Cardiogenic shock | 1268132 | 54 | 85491 | 7 | 0.96(0.43-2.13) | -0.00 (-0.02, 0.01) |
| Pulmonary embolism | 1267634 | 162 | 85451 | 13 | 0.79(0.43-1.43) | -0.03 (-0.05, -0.00) |
| Deep venous thrombosis | 1266716 | 323 | 85343 | 41 | 1.24(0.85-1.80) | 0.06 (0.02, 0.10) |
| Superficial venous thromboses | 1267932 | 56 | 85467 | 9 | 2.54(1.08-5.95) | 0.07 (0.05, 0.09) |
| Arterial thromboses | 1268056 | 27 | 85489 | 1 | 0.19(0.03-1.40) | -0.02 (-0.03, -0.01) |
| Pericarditis | 1268114 | 11 | 85489 | 3 | 3.56(0.92-13.70) | 0.02 (0.01, 0.03) |
| Myocarditis | 1268169 | 10 | 85498 | 0 | –^#^ | -0.01 (-0.01, -0.00) |

Hazards ratio (HR) and excess burdens of pre-specified cardiovascular complications in the COVID-19 and control groups. 95% confidence intervals are reported in parentheses.

*denotes 95% CIs which are bounded away from 0. ^#^not estimable due to 0 cases in either group.

HR > 1 denotes higher risk of a respective composite/individual new cardiovascular condition in the COVID-19 exposed group versus control group.

Excess burdens > 0 denotes excess burden in a respective composite/individual new cardiovascular condition in the COVID-19 exposed group versus control group

# Table 16: Subgroup analysis with inverse probability weights without covariates in regression specification: hazards ratios and excess burdens per 1000 person years for fully vaccinated and boosted

|  | No. of Individuals | | | |  |  |
| --- | --- | --- | --- | --- | --- | --- |
| Outcome | Controls | Controls with outcome | Cases | Cases with outcome | HR (no cov) | Excess burden (weighted, per 1000 person years) |
| Any cardiovascular, cerebrovascular  and other thrombotic complications | 278671 | 3221 | 10327 | 153 | 1.10(0.92-1.31) | 1.11 (0.55, 1.68) |
| MACE | 294598 | 1857 | 11005 | 83 | 1.04(0.81-1.34) | 0.27 (-0.13, 0.68) |
| Cerebrovascular disorders | 293208 | 1076 | 10994 | 55 | 1.20(0.89-1.62) | 0.75 (0.43, 1.07) |
| Dysrhythmias | 297408 | 921 | 11182 | 52 | 1.36(0.99-1.88) | 1.12 (0.82, 1.42) |
| Ischemic heart diseases | 292101 | 1479 | 10915 | 67 | 0.98(0.75-1.29) | -0.10 (-0.46, 0.26) |
| Other cardiac disorders | 300594 | 621 | 11297 | 23 | 0.92(0.59-1.44) | -0.16 (-0.38, 0.06) |
| Thrombotic disorders | 301837 | 217 | 11358 | 8 | 0.87(0.42-1.81) | -0.10 (-0.22, 0.03) |
| Inflammatory heart disease | 302691 | 4 | 11403 | 0 | –^#^ | -0.01 (-0.03, -0.00) |
| Stroke | 294248 | 904 | 11037 | 43 | 1.13(0.80-1.59) | 0.39 (0.10, 0.68) |
| TIA | 301084 | 254 | 11336 | 14 | 1.25(0.72-2.18) | 0.21 (0.06, 0.37) |
| Atrial fibrillation | 300018 | 517 | 11291 | 22 | 1.06(0.64-1.74) | 0.10 (-0.11, 0.31) |
| Sinus tachycardia | 301946 | 149 | 11361 | 6 | 0.94(0.40-2.20) | -0.03 (-0.14, 0.08) |
| Sinus bradycardia | 302190 | 74 | 11380 | 5 | 1.39(0.55-3.55) | 0.10 (0.01, 0.18) |
| Other arrhythmia | 300883 | 286 | 11340 | 26 | 2.19(1.39-3.46) | 1.13 (0.94, 1.32) |
| Myocardial infarction | 299124 | 785 | 11227 | 44 | 1.24(0.88-1.76) | 0.64 (0.37, 0.91) |
| Acute coronary disease | 295172 | 1007 | 11063 | 42 | 0.86(0.63-1.20) | -0.46 (-0.75, -0.18) |
| Ischemic cardiomyopathy | 302387 | 70 | 11391 | 5 | 1.55(0.61-3.93) | 0.13 (0.04, 0.22) |
| Angina | 299158 | 260 | 11235 | 11 | 0.83(0.44-1.54) | -0.15 (-0.29, -0.01) |
| Heart failure | 300966 | 514 | 11317 | 14 | 0.70(0.39-1.26) | -0.51 (-0.70, -0.32) |
| Non ischemic cardiomyopathy | 302225 | 88 | 11381 | 7 | 1.79(0.81-3.94) | 0.23 (0.13, 0.33) |
| Cardiac arrest | 302700 | 55 | 11400 | 3 | 1.55(0.46-5.30) | 0.10 (0.03, 0.18) |
| Cardiogenic shock | 302681 | 21 | 11404 | 0 | –^#^ | -0.07 (-0.10, -0.04) |
| Pulmonary embolism | 302475 | 70 | 11396 | 5 | 1.74(0.67-4.51) | 0.17 (0.08, 0.26) |
| Deep venous thrombosis | 302145 | 136 | 11372 | 3 | 0.45(0.14-1.45) | -0.25 (-0.34, -0.16) |
| Superficial venous thromboses | 302613 | 25 | 11402 | 0 | –^#^ | -0.08 (-0.11, -0.05) |
| Arterial thromboses | 302644 | 12 | 11399 | 1 | 1.78(0.23-13.70) | 0.03 (-0.01, 0.07) |
| Pericarditis | 302700 | 2 | 11404 | 0 | –^#^ | -0.01 (-0.02, 0.00) |
| Myocarditis | 302708 | 2 | 11404 | 0 | –^#^ | -0.01 (-0.02, 0.00) |

Hazards ratio (HR) and excess burdens of pre-specified cardiovascular complications in the COVID-19 and control groups. 95% confidence intervals are reported in parentheses.

*denotes 95% CIs which are bounded away from 0. ^#^not estimable due to 0 cases in either group.

HR > 1 denotes higher risk of a respective composite/individual new cardiovascular condition in the COVID-19 exposed group versus control group.

Excess burdens > 0 denotes excess burden in a respective composite/individual new cardiovascular condition in the COVID-19 exposed group versus control group

# Table 17: Subgroup analysis with inverse probability weights without covariates in regression specification: hazards ratios and excess burdens per 1000 person years for severe cases

|  | No. of Individuals | | | |  |  |
| --- | --- | --- | --- | --- | --- | --- |
| Outcome | Controls | Controls with outcome | Cases | Cases with outcome | HR (no cov) | Excess burden (weighted, per 1000 person years) |
| Any cardiovascular, cerebrovascular  and other thrombotic complications | 1607157 | 9970 | 1633 | 82 | 5.47(3.41-8.78) | 27.34 (26.93, 27.75) |
| MACE | 1656164 | 5657 | 1895 | 63 | 6.82(4.24-10.96) | 19.73 (19.39, 20.06) |
| Cerebrovascular disorders | 1655044 | 3276 | 1920 | 23 | 2.03(0.98-4.22) | 2.05 (1.89, 2.20) |
| Dysrhythmias | 1664562 | 3071 | 2026 | 34 | 4.65(2.53-8.55) | 6.74 (6.53, 6.96) |
| Ischemic heart diseases | 1652224 | 4391 | 1967 | 40 | 8.16(4.51-14.76) | 18.88 (18.56, 19.21) |
| Other cardiac disorders | 1676187 | 1895 | 2034 | 35 | 8.32(4.99-13.87) | 8.30 (8.08, 8.51) |
| Thrombotic disorders | 1680478 | 809 | 2104 | 10 | 2.26(1.01-5.02) | 0.61 (0.53, 0.69) |
| Inflammatory heart disease | 1683885 | 23 | 2151 | 0 | –^#^ | -0.01 (-0.02, -0.01) |
| Stroke | 1658327 | 2776 | 1934 | 22 | 2.37(1.13-4.96) | 2.29 (2.14, 2.44) |
| TIA | 1678949 | 733 | 2128 | 2 | 0.31(0.07-1.33) | -0.30 (-0.34, -0.26) |
| Atrial fibrillation | 1675638 | 1521 | 2074 | 17 | 2.36(1.07-5.21) | 1.24 (1.13, 1.35) |
| Sinus tachycardia | 1680493 | 652 | 2126 | 8 | 3.78(1.43-9.99) | 1.08 (0.99, 1.17) |
| Sinus bradycardia | 1682277 | 272 | 2140 | 6 | 7.01(2.64-18.61) | 0.97 (0.90, 1.05) |
| Other arrhythmia | 1676468 | 1006 | 2115 | 10 | 7.14(2.61-19.57) | 3.69 (3.55, 3.84) |
| Myocardial infarction | 1671974 | 2454 | 2069 | 26 | 9.39(4.83-18.23) | 12.28 (12.03, 12.54) |
| Acute coronary disease | 1661961 | 2882 | 2042 | 16 | 6.05(2.46-14.84) | 8.72 (8.49, 8.95) |
| Ischemic cardiomyopathy | 1682982 | 228 | 2131 | 2 | 29.53(7.19-121.21) | 3.91 (3.77, 4.04) |
| Angina | 1673700 | 802 | 2091 | 4 | 1.82(0.44-7.60) | 0.39 (0.32, 0.47) |
| Heart failure | 1677494 | 1524 | 2043 | 29 | 8.82(5.07-15.34) | 7.13 (6.93, 7.33) |
| Non ischemic cardiomyopathy | 1682194 | 297 | 2136 | 4 | 1.41(0.38-5.15) | 0.07 (0.03, 0.11) |
| Cardiac arrest | 1683998 | 188 | 2150 | 3 | 8.72(2.28-33.28) | 0.87 (0.80, 0.94) |
| Cardiogenic shock | 1683937 | 81 | 2150 | 2 | 14.29(2.69-75.83) | 0.64 (0.58, 0.70) |
| Pulmonary embolism | 1683139 | 257 | 2139 | 3 | 25.41(3.93-164.30) | 3.74 (3.60, 3.87) |
| Deep venous thrombosis | 1681727 | 509 | 2117 | 8 | 2.91(1.17-7.26) | 0.58 (0.51, 0.65) |
| Superficial venous thromboses | 1683640 | 88 | 2149 | 1 | 4.18(0.58-30.14) | 0.17 (0.13, 0.20) |
| Arterial thromboses | 1683809 | 47 | 2147 | 0 | –^#^ | -0.03 (-0.04, -0.02) |
| Pericarditis | 1683951 | 13 | 2151 | 0 | –^#^ | -0.01 (-0.01, -0.00) |
| Myocarditis | 1684013 | 12 | 2151 | 0 | –^#^ | -0.01 (-0.01, -0.00) |

Hazards ratio (HR) and excess burdens of pre-specified cardiovascular complications in the COVID-19 and control groups. 95% confidence intervals are reported in parentheses.

*denotes 95% CIs which are bounded away from 0. ^#^not estimable due to 0 cases in either group.

HR > 1 denotes higher risk of a respective composite/individual new cardiovascular condition in the COVID-19 exposed group versus control group.

Excess burdens > 0 denotes excess burden in a respective composite/individual new cardiovascular condition in the COVID-19 exposed group versus control group

# Table 18: Subgroup analysis with inverse probability weights without covariates in regression specification: hazards ratios and excess burdens per 1000 person years for hospitalised cases

|  | No. of Individuals | | | |  |  |
| --- | --- | --- | --- | --- | --- | --- |
| Outcome | Controls | Controls with outcome | Cases | Cases with outcome | HR (no cov) | Excess burden (weighted, per 1000 person years) |
| Any cardiovascular, cerebrovascular  and other thrombotic complications | 1607157 | 9970 | 6553 | 206 | 2.89(2.10-4.00) | 11.76 (11.51, 12.00) |
| MACE | 1656164 | 5657 | 7523 | 151 | 2.06(1.36-3.13) | 3.65 (3.49, 3.81) |
| Cerebrovascular disorders | 1655044 | 3276 | 7617 | 61 | 3.03(1.52-6.04) | 4.05 (3.91, 4.19) |
| Dysrhythmias | 1664562 | 3071 | 7920 | 76 | 2.95(1.80-4.85) | 3.64 (3.51, 3.78) |
| Ischemic heart diseases | 1652224 | 4391 | 7695 | 90 | 1.57(1.12-2.20) | 1.53 (1.40, 1.66) |
| Other cardiac disorders | 1676187 | 1895 | 8050 | 77 | 2.89(1.96-4.27) | 2.18 (2.07, 2.28) |
| Thrombotic disorders | 1680478 | 809 | 8283 | 34 | 4.19(2.33-7.53) | 1.56 (1.48, 1.64) |
| Inflammatory heart disease | 1683885 | 23 | 8411 | 0 | –^#^ | -0.01 (-0.02, -0.01) |
| Stroke | 1658327 | 2776 | 7676 | 51 | 2.40(1.14-5.06) | 2.37 (2.26, 2.49) |
| TIA | 1678949 | 733 | 8298 | 14 | 4.52(1.10-18.62) | 1.55 (1.47, 1.63) |
| Atrial fibrillation | 1675638 | 1521 | 8110 | 43 | 1.88(1.12-3.14) | 0.81 (0.73, 0.89) |
| Sinus tachycardia | 1680493 | 652 | 8342 | 17 | 1.97(0.88-4.39) | 0.38 (0.33, 0.43) |
| Sinus bradycardia | 1682277 | 272 | 8368 | 9 | 7.46(1.70-32.83) | 1.06 (1.00, 1.12) |
| Other arrhythmia | 1676468 | 1006 | 8272 | 26 | 3.37(1.54-7.34) | 1.43 (1.35, 1.51) |
| Myocardial infarction | 1671974 | 2454 | 8077 | 61 | 1.45(0.89-2.35) | 0.66 (0.57, 0.76) |
| Acute coronary disease | 1661961 | 2882 | 7999 | 50 | 1.62(1.07-2.48) | 1.09 (0.98, 1.19) |
| Ischemic cardiomyopathy | 1682982 | 228 | 8356 | 6 | 2.03(0.65-6.29) | 0.14 (0.11, 0.18) |
| Angina | 1673700 | 802 | 8174 | 20 | 1.98(1.10-3.55) | 0.47 (0.41, 0.53) |
| Heart failure | 1677494 | 1524 | 8103 | 70 | 2.93(1.93-4.46) | 1.79 (1.70, 1.88) |
| Non ischemic cardiomyopathy | 1682194 | 297 | 8335 | 8 | 1.75(0.59-5.19) | 0.13 (0.10, 0.17) |
| Cardiac arrest | 1683998 | 188 | 8409 | 5 | 3.05(0.73-12.77) | 0.24 (0.20, 0.27) |
| Cardiogenic shock | 1683937 | 81 | 8410 | 1 | 0.35(0.05-2.51) | -0.03 (-0.04, -0.02) |
| Pulmonary embolism | 1683139 | 257 | 8387 | 6 | 2.79(1.05-7.41) | 0.28 (0.24, 0.31) |
| Deep venous thrombosis | 1681727 | 509 | 8315 | 28 | 5.14(2.53-10.43) | 1.27 (1.21, 1.34) |
| Superficial venous thromboses | 1683640 | 88 | 8404 | 1 | 1.20(0.17-8.62) | 0.01 (-0.01, 0.03) |
| Arterial thromboses | 1683809 | 47 | 8406 | 2 | 1.41(0.26-7.69) | 0.01 (-0.00, 0.02) |
| Pericarditis | 1683951 | 13 | 8411 | 0 | –^#^ | -0.01 (-0.01, -0.00) |
| Myocarditis | 1684013 | 12 | 8414 | 0 | –^#^ | -0.01 (-0.01, -0.00) |

Hazards ratio (HR) and excess burdens of pre-specified cardiovascular complications in the COVID-19 and control groups. 95% confidence intervals are reported in parentheses.

*denotes 95% CIs which are bounded away from 0. ^#^not estimable due to 0 cases in either group.

HR > 1 denotes higher risk of a respective composite/individual new cardiovascular condition in the COVID-19 exposed group versus control group.

Excess burdens > 0 denotes excess burden in a respective composite/individual new cardiovascular condition in the COVID-19 exposed group versus control group

# Table 19: Subgroup analysis with inverse probability weights without covariates in regression specification: hazards ratios and excess burdens per 1000 person years for mild cases

|  | No. of Individuals | | | |  |  |
| --- | --- | --- | --- | --- | --- | --- |
| Outcome | Controls | Controls with outcome | Cases | Cases with outcome | HR (no cov) | Excess burden (weighted, per 1000 person years) |
| Any cardiovascular, cerebrovascular  and other thrombotic complications | 1607157 | 9970 | 91028 | 624 | 0.95(0.86-1.04) | -0.33 (-0.50, -0.16) |
| MACE | 1656164 | 5657 | 93797 | 346 | 0.89(0.79-1.01) | -0.38 (-0.50, -0.26) |
| Cerebrovascular disorders | 1655044 | 3276 | 93902 | 206 | 0.97(0.83-1.13) | -0.07 (-0.16, 0.03) |
| Dysrhythmias | 1664562 | 3071 | 94462 | 201 | 1.11(0.94-1.30) | 0.20 (0.10, 0.29) |
| Ischemic heart diseases | 1652224 | 4391 | 93421 | 262 | 0.88(0.77-1.02) | -0.31 (-0.42, -0.21) |
| Other cardiac disorders | 1676187 | 1895 | 95048 | 124 | 0.95(0.77-1.17) | -0.06 (-0.13, 0.01) |
| Thrombotic disorders | 1680478 | 809 | 95275 | 40 | 0.80(0.56-1.14) | -0.10 (-0.14, -0.06) |
| Inflammatory heart disease | 1683885 | 23 | 95432 | 3 | 1.68(0.48-5.91) | 0.01 (0.00, 0.02) |
| Stroke | 1658327 | 2776 | 94078 | 170 | 0.91(0.77-1.08) | -0.15 (-0.24, -0.07) |
| TIA | 1678949 | 733 | 95158 | 47 | 1.07(0.77-1.50) | 0.03 (-0.01, 0.08) |
| Atrial fibrillation | 1675638 | 1521 | 95075 | 82 | 0.90(0.69-1.16) | -0.10 (-0.16, -0.03) |
| Sinus tachycardia | 1680493 | 652 | 95248 | 33 | 0.81(0.55-1.19) | -0.07 (-0.11, -0.03) |
| Sinus bradycardia | 1682277 | 272 | 95351 | 24 | 1.27(0.80-2.04) | 0.04 (0.02, 0.07) |
| Other arrhythmia | 1676468 | 1006 | 95033 | 82 | 1.48(1.15-1.92) | 0.29 (0.23, 0.35) |
| Myocardial infarction | 1671974 | 2454 | 94627 | 148 | 0.89(0.73-1.08) | -0.16 (-0.24, -0.09) |
| Acute coronary disease | 1661961 | 2882 | 93995 | 172 | 0.86(0.72-1.02) | -0.25 (-0.34, -0.17) |
| Ischemic cardiomyopathy | 1682982 | 228 | 95379 | 13 | 0.87(0.47-1.63) | -0.02 (-0.04, 0.01) |
| Angina | 1673700 | 802 | 94773 | 63 | 1.13(0.85-1.51) | 0.06 (0.02, 0.11) |
| Heart failure | 1677494 | 1524 | 95124 | 89 | 0.83(0.65-1.06) | -0.16 (-0.22, -0.10) |
| Non ischemic cardiomyopathy | 1682194 | 297 | 95342 | 31 | 1.56(1.05-2.32) | 0.10 (0.07, 0.13) |
| Cardiac arrest | 1683998 | 188 | 95441 | 11 | 0.90(0.46-1.76) | -0.01 (-0.03, 0.01) |
| Cardiogenic shock | 1683937 | 81 | 95440 | 5 | 0.60(0.24-1.49) | -0.02 (-0.03, -0.01) |
| Pulmonary embolism | 1683139 | 257 | 95406 | 12 | 0.73(0.39-1.36) | -0.04 (-0.07, -0.02) |
| Deep venous thrombosis | 1681727 | 509 | 95335 | 20 | 0.59(0.36-0.96) | -0.12 (-0.16, -0.09) |
| Superficial venous thromboses | 1683640 | 88 | 95417 | 8 | 1.70(0.73-3.98) | 0.04 (0.02, 0.05) |
| Arterial thromboses | 1683809 | 47 | 95437 | 1 | 0.58(0.08-4.23) | -0.01 (-0.02, -0.00) |
| Pericarditis | 1683951 | 13 | 95436 | 3 | 2.96(0.80-10.95) | 0.02 (0.01, 0.02) |
| Myocarditis | 1684013 | 12 | 95443 | 0 | –^#^ | -0.01 (-0.01, -0.00) |

Hazards ratio (HR) and excess burdens of pre-specified cardiovascular complications in the COVID-19 and control groups. 95% confidence intervals are reported in parentheses.

*denotes 95% CIs which are bounded away from 0. ^#^not estimable due to 0 cases in either group.

HR > 1 denotes higher risk of a respective composite/individual new cardiovascular condition in the COVID-19 exposed group versus control group.

Excess burdens > 0 denotes excess burden in a respective composite/individual new cardiovascular condition in the COVID-19 exposed group versus control group

# Table 20: Negative outcome controls: hazards ratios and excess burdens per 1000 person years

|  | No. of Individuals | | | |  |  |
| --- | --- | --- | --- | --- | --- | --- |
| Outcome | Controls | Controls with outcome | Cases | Cases with outcome | HR | Excess burden (weighted, per 1000 persons) |
| Bcell Lymphoma | 1683074 | 124 | 105902 | 15 | 1.15 (0.65,2.01) | 0.01 (-0.01, 0.03) |
| Hodgkins Lymphoma | 1683854 | 16 | 105986 | 1 | 0.89(0.12-6.67) | -0.00 (-0.01, 0.00) |
| Malignancy (Tongue) | 1683855 | 35 | 105995 | 3 | 0.95(0.27-3.33) | -0.00 (-0.01, 0.01) |
| Atopic Dermatitis | 1682763 | 119 | 105903 | 8 | 1.24(0.57-2.71) | 0.02 (0.00, 0.04) |
| Composite outcome (negative outcomes | 1681323 | 291 | 105754 | 27 | 1.15(0.72-1.82) | 0.03 (-0.00, 0.05) |

Hazards ratio (HR) and excess burdens of pre-specified negative outcome controls in the COVID-19 and control groups. 95% confidence intervals are reported in parentheses.

*denotes 95% CIs which are bounded away from 0. ^#^not estimable due to 0 myocarditis cases in COVID-19 survivor group.

HR > 1 denotes higher risk of a respective composite/individual negative outcomes in the COVID-19 exposed group versus control group.

Excess burdens > 0 denotes excess burden in a respective composite/individual new negative outcomes in the COVID-19 exposed group versus control group
